# Global patterns of asthma burden related to environmental risk factors during 1990–2019: an age-period-cohort analysis for global burden of disease study 2019

**DOI:** 10.1186/s12940-024-01060-8

**Published:** 2024-02-15

**Authors:** Siying Zhang, Zongshi Gao, Lihong Wu, Yumei Zhong, Hui Gao, Fang-biao Tao, Xiulong Wu

**Affiliations:** 1 Department of Maternal, Child and Adolescent Health, School of Public Health, Anhui Medical University, No 81 Meishan Road, Hefei, Anhui 230032 China; 2 Key Laboratory of Population Health Across Life Cycle (Anhui Medical University), Ministry of Education of the People’s Republic of China, No 81 Meishan Road, Hefei, Anhui 230032 China; 3 Anhui Provincial Key Laboratory of Environment and Population Health across the Life Course, Anhui Medical University, No 81 Meishan Road, Hefei, Anhui 230032 China; 4 The First Clinical College of Anhui Medical University, Anhui Medical University, No 81 Meishan Road, Hefei, Anhui 230032 China; 5 Department of Pediatrics, The First Affiliated Hospital of Anhui Medical University, No 218 Jixi Road, Hefei, Anhui 230022 China

**Keywords:** Asthma, Disability-adjusted life years, Smoking, Occupational asthmagens, Age-period-cohort analysis

## Abstract

**Background:**

Change in asthma burden attributed to specific environmental risk factor has not been evaluated.

**Objective:**

We aimed to explore the age, period, and cohort effects on asthma burden attributable to smoking and occupational asthmagens in different socio-demographic index (SDI) regions and the region and sex disparities.

**Methods:**

Risk factor-specific asthma deaths and disability-adjusted life years (DALYs) rates were extracted from Global Burden of Disease study 2019, estimated by standard Combined Cause of Death Model and DisMod-MR 2.1 modeling tool. Age-period-cohort analysis was conducted to decompose age, period, and cohort effects on asthma burden.

**Results:**

Smoking- and occupational asthmagens-related asthma deaths and DALYs rates dropped by > 45% during 1990–2019. In 2019, Africa, South and Southeast Asia had higher asthma burden than other regions. Male had higher asthma burden than female. Among nearly all age groups, low-middle SDI region had the highest smoking-related asthma burden, and low SDI region had the highest occupational asthmagens-related asthma burden. Inverse “V” shaped trend was observed in the above regions with increasing age. For smoking-related asthma deaths and DALYs rates, the most significant improvement of period rate ratio (RR) occurred in high SDI region, decreased from 1.67 (1.61, 1.74) to 0.34 (0.33, 0.36) and 1.61 (1.57, 1.66) to 0.59 (0.57, 0.61), respectively, as well as the cohort effect on smoking-related asthma burden. For occupational asthmagens-related asthma deaths and DALYs rates, the most sharply decrease of period and cohort RR appeared in the high and high-middle SDI regions. Low SDI region showed least progress in period and cohort RR of smoking- and occupational asthmagens-linked asthma burden.

**Conclusion:**

Smoking- and occupational asthmagens-related asthma burden sharply decreases, but region and sex disparities exist. Policy makers from low SDI region should reinforce tobacco control and prioritize workplace protection.

**Supplementary Information:**

The online version contains supplementary material available at 10.1186/s12940-024-01060-8.

## Introduction

Asthma is a common chronic respiratory disease, especially in children and elders, leading to 461.1 thousand deaths and 21.6 million disability-adjusted life years (DALYs) in 2019 [1]. Prevalence of asthma in children was higher in developed countries than developing countries [2, 3], but most asthma-related deaths occurred in low- and middle-income countries. Benefiting from the use of inhaled corticosteroids, asthma mortality decreased over the last few decades worldwide. However, asthma-related years of life lost (YLLs) in the low and low-middle socio-demographic index (SDI) regions remained well ahead of other regions [4]. Although asthma prevalence was low in the Sub-Saharan Africa and South Asia, these areas have higher asthma DALYs rates due to high risk of asthma deaths than other regions [5]. Relationship between SDI and asthma DALYs rate for all ages showed a decreasing trend as the SDI-level increasing [1]. Besides, there is sex disparity in asthma burden due to differences in environmental exposures, including smoking and occupational exposure [6].

According to the Global Burden of Disease (GBD) study 2019 on asthma, smoking and occupational asthmagens contributed 11.93% and 7.47% to the global asthma deaths, respectively; 9.9% and 8.8% to the global asthma DALYs, respectively [1]. Smoking is an important risk factor for asthma, and the causal association has been well documented [7]. High SDI regions have achieved outstanding tobacco control, but in the lower SDI countries, tobacco industry activity increased aggressively and the smoking rate rose rapidly [8]. Smoking causes corticosteroid insensitivity, which could affect asthma control. In addition, smokers with asthma have the accelerated decline of lung function and higher prevalence of comorbidities, further increased asthma burden [9]. Approximately 25-50% of occupational asthma had exacerbated respiratory symptoms associated with exposure to asthmagens in the workplace [10], like low-molecular-height compounds (mostly isocyanates) and high-molecular-weight agents (mostly flour) [11]. Difficulties in the diagnosis, poor awareness of occupational protection, and absence of well-defined occupational exposure limits led to underestimation and underreporting of occupational asthma [12, 13]. Recently, Safiri et al. analyzed the global epidemic pattern of asthma, but age, period, and cohort effects were not clarified, as well as the changes in contribution of specific risk factor [1]. Bai et al. conducted an age-period-cohort (APC) analysis of asthma deaths only in four countries (Brazil, Russia, China, and South Africa), global pattern of asthma DALYs was not evaluated, and they also did not perform risk factor-specific analyses [14].

This study aimed to evaluate the updated global patterns of asthma deaths and DALYs rates related to different environmental risk factors during 1990–2019. GBD 2019 used the standard Combined Cause of Death Model (CODEm) approach and the DisMod-MR 2.1 modeling tool to estimate risk factors attributable asthma deaths and DALYs rates at the global, regional, and country levels. The APC model was used to explore changes in the risk factor-specific asthma burden associated with age, period, and cohort effects in the worldwide and five SDI regions, which would help to evaluate the difference in the improvement of asthma burden across different SDI regions.

## Methods

### Data sources

Data used in this study are from the GBD 2019 Study, which estimates the incidence, prevalence, deaths, and DALYs for 369 diseases and injuries in 204 countries and territories, and provides disease burden attributed to 87 risk factors [15]. Multi-source data were used to evaluate the distribution of asthma burden by age, sex, region, and year, including censuses, disease registries, epidemiological surveys, health service use, vital statistics, disease and risk factors surveillance systems, air pollution monitors, and other sources [16]. Detailed data sources are available on the GBD 2019 data input sources tool (https://ghdx.healthdata.org/gbd-2019/data-input-sources). The World Cancer Research Fund criteria have been used to evaluate credible or possible proof of risk-asthma pairs since 2010, and only the pairs with strong evidence were included. Asthma deaths and DALYs rates ascribed to smoking and occupational asthmagens were available from GBD 2019 database. Risk factor-specific asthma burden was explored in the whole world and countries and territories of different SDIs. Components of SDI included income per capita, the fertility rate in females under 25 years old, and average years of schooling for individuals aged 15 years and above. All the countries and territories were divided into quintiles based on SDI, including low, low-middle, middle, high-middle, and high SDI (SDI quintiles were listed in Table S1, and countries and territories of different SDI groups were included in Table S2) [16, 17].

### Definition and estimation of asthma burden

Asthma is defined as a doctor’s diagnosis and wheezing in the past year, and it is coded by the *International Statistical Classification of Diseases* and *Related Health Problems* (J45-J46 in ICD-10, and 493 in ICD-9). There were four alternative asthma definitions: self-reported asthma in the past year, self-reported lifetime asthma, only a doctor’s diagnosis in the past year, and only wheezing in the past year.

To estimate deaths due to asthma, GBD 2019 applied the CODEm approach. Death models were established for both sexes and age groups ranging from 1 to over 95 years old. DisMod-MR 2.1 was the main modeling tool for asthma and maximum remission was set as 0.3 (revealing the upper bound of the largest observed data) and no data for 0–0.5 age group because the diagnosis of asthma in young infants was unable. Log lag distributed income, asthma standardized exposure variables, healthcare services and quality index were included in the DisMod model as covariates to improve comprehensiveness and robustness of estimation (Table S3). The DALYs are the sum of years lived with disability and YLLs due to premature deaths. The proportion of asymptomatic (36.2%), controlled (19.9%), partially controlled (20.6%) and uncontrolled asthma (23.3%) was estimated using data from USA Medical Expenditure Panel Surveys during 2000–2014, and the disability weights were assigned to 0, 0.015, 0.036, and 0.133, respectively (Table S4). Based on the population size and asthma prevalence of different SDI subgroups, total and severity-specific numbers of asthma cases were calculated, and then severity-specific disability weights were assigned to calculate the number of years lived with disability with comorbidity correction. Sex-specific deaths rates in the different age groups were estimated by using CODEm and then multiplied by the number of people in each age groups to obtain the number of deaths, and YLLs could be calculated according to the life expectancy of the subgroup in the standard life table. In the maps showing age-standardized asthma deaths and DALYs rates, 204 countries and territories were stratified by sextile of risk factor-specific deaths and DALYs rates of asthma, respectively.

### Definitions of risk factors

Smokers included current smokers and former smokers. Current smokers were the individuals who occasionally or daily smoked, and former smokers were these who have quitted tobacco products for at least six months. Data for occupational risks were from the International Labor Organization, in which 22 occupational asthmagens were recorded (Table S5). Proportion of exposure to asthmagens of workers was estimated according to the population distribution of nine occupational groups [15].

### Statistical analysis

APC model was used to explore the age, period, and cohort effects on the asthma deaths and DALYs rates attributed to smoking and occupational asthmagens. Age effect showed variation in asthma burden with increasing age. Period effect represented the influence of technological factors on asthma burden, including screening method, disease definition, and treatment, which would affect all age groups. And cohort effect indicated the difference in asthma burden across different birth cohorts [18]. APC model expresses as follows:

$$\mathrm{Ln}\;({\mathrm Y}_{ij})\;=\;\mu\:+\:\alpha_i\:+\:\beta_j\:+\:\gamma_k\\$$

Here Y_*ij*_ represents risk factors-specific rates of asthma deaths and DALYs, *α*_*i*_ shows the age effect in the age group *i*, *β*_*j*_ represents the period effect in the period group *j*, γ_*k*_ indicates the risk factors-specific rate ratio (RR) of asthma in the birth cohort *k* related to age group *i* and period group *j* (*k* = *j*−*i*), *µ* denotes the intercept.

An online tool for APC analysis was provided by the National Institutes of Health (https://analysistools.cancer.gov/apc/) [18], and risk factors specific-asthma rates of deaths and DALYs were assumed to follow Poisson distribution. Intrinsic estimator was applied to address multicollinearity problem, which was built on singular value decomposition of matrices. Local drifts reflected annual percent change of age group-specific rates over time, and net drifts reflected overall annual percent change. Age and period were both divided by 5-year interval, and there were 13 (30–34 to 90–94) age groups for smoking-related asthma deaths and DALYs rates and 14 (15–19 to 80–84) age groups for occupational asthmagens. Six period groups were from 1990–1994 to 2015–2019. Birth cohort = period−age, 18 (1900–1904 to 1985–1989) birth cohorts were produced for smoking-related asthma deaths and DALYs rates and 19 (1910–1914 to 2000–2004) birth cohorts for occupational asthmagens. The medians of period groups (2000–2004) and birth cohorts (1940–1944 for smoking; 1955–1959 for occupational asthmagens) were defined as reference, and RR of risk factors attributable asthma deaths and DALYs rates in the given period and birth cohort was calculated. Two-sided *P* < 0.05 was regarded as statistically significant.

## Results

### Change of asthma burden during 1990–2019

Global deaths from asthma attributable to smoking and occupational asthmagens were 54,850 [95% *uncertainty interval* (*UI*) = (29,150, 78,010)] and 34,400 [95% *UI* = (27,830, 42,610)] cases in 2019, respectively (Table S6). Age-standardized deaths percent of asthma related to smoking and occupational asthmagens declined by 23.01% and 7.79% (Table S7). Percent changes for smoking- and occupational asthmagens-related asthma deaths rate were −62.43% (declined from 1.81 per 100,000 in 1990 to 0.68 per 100,000 in 2019) and −55.43% (declined from 0.92 per 100,000 in 1990 to 0.41 per 100,000 in 2019), respectively. The drop of asthma deaths rate attributable to each risk factor was higher in male than that in female. Furthermore, smoking- and occupational asthmagens-related asthma deaths rates experienced significant declines in all SDI regions during 1990–2019 (Table 1).

Global DALYs numbers of asthma attributable to smoking and occupational asthmagens were 2.12 [95% *UI* = (1.13, 3.01)] million and 1.90 [95% *UI* = (1.51, 2.33)] million in 2019, respectively (Table S8). Age-standardized DALYs percent of asthma related to smoking and occupational asthmagens declined by 29.52% and 5.62%, respectively (Table S9). Percent changes for smoking- and occupational asthmagens-related asthma DALYs rate were −59.60% (declined from 63.21 per 100,000 in 1990 to 25.54 per 100,000 in 2019) and −45.93% (declined from 42.35 per 100,000 in 1990 to 22.9 per 100,000 in 2019), respectively. Compared with female, improvement of asthma DALYs rates attributable to each risk factor was greater in male. Moreover, smoking- and occupational asthmagens-related asthma DALYs rates showed effective descents in all SDI regions during 1990–2019 (Table 2).

**Table 1:** Change in age-standardized asthma deaths rate related to environmental risk factors from 1990 to 2019

Categories	Smoking-related asthma deaths rate (per 100,000 population)			Occupational asthmagens-related asthma deaths rate (per 100,000 population)		
	1990	2019	Percent change	1990	2019	Percent change
Global	1.81 (0.94, 2.74)	0.68 (0.36, 0.96)	−62.43%	0.92 (0.65, 1.26)	0.41 (0.33, 0.51)	−55.43%
Sex						
Male	3.44 (1.77, 5.21)	1.21 (0.65, 1.70)	−64.83%	1.37 (0.90, 1.98)	0.60 (0.45, 0.76)	−56.20%
Female	0.56 (0.28, 0.93)	0.23 (0.11, 0.37)	−58.93%	0.51 (0.32, 0.77)	0.24 (0.18, 0.31)	−52.94%
SDI region						
High SDI	0.77 (0.44, 1.10)	0.14 (0.08, 0.21)	−81.82%	0.19 (0.18, 0.21)	0.06 (0.05, 0.07)	−68.42%
High-middle SDI	0.95 (0.54, 1.35)	0.24 (0.13, 0.33)	−74.74%	0.29 (0.25, 0.36)	0.08 (0.07, 0.10)	−72.41%
Middle SDI	1.95 (1.04, 2.90)	0.71 (0.39, 0.99)	−63.59%	0.78 (0.62, 1.04)	0.31 (0.26, 0.36)	−60.26%
Low-middle SDI	4.98 (2.37, 8.26)	1.90 (0.95, 2.86)	−61.85%	2.54 (1.63, 3.76)	1.04 (0.76, 1.40)	−59.06%
Low SDI	3.37 (1.49, 5.77)	1.60 (0.75, 2.57)	−52.52%	3.08 (2.12, 4.47)	1.47 (1.11, 2.06)	−52.27%

*Abbreviation:* *SDI* Socio-demographic index

**Table 2:** Change in age-standardized asthma DALYs rate related to environmental risk factors from 1990 to 2019

Categories	Smoking-related asthma DALYs rate (per 100,000 population)			Occupational asthmagens-related asthma DALYs rate (per 100,000 population)		
	1990	2019	Percent change	1990	2019	Percent change
Global	63.21 (34.83, 90.44)	25.54 (13.57, 36.17)	−59.60%	42.35 (33.02, 53.52)	22.90 (18.21, 28.21)	−45.93%
Sex						
Male	103.30 (56.27, 147.54)	40.29 (21.87, 56.08)	−61.00%	58.19 (43.04, 75.79)	30.25 (23.86, 37.37)	−48.02%
Female	28.90 (14.49, 43.18)	12.15 (6.02, 18.37)	−57.96%	27.42 (20.32, 35.61)	15.82 (12.28, 20.31)	−42.30%
SDI region						
High SDI	67.10 (35.22, 100.67)	27.82 (13.57, 43.87)	−58.54%	32.98 (23.73, 45.11)	21.79 (14.87, 30.98)	−33.93%
High-middle SDI	42.93 (24.04, 59.99)	15.23 (8.00, 22.26)	−64.52%	21.77 (16.84, 28.12)	10.60 (7.70, 14.63)	−51.31%
Middle SDI	50.22 (27.82, 71.96)	21.55 (11.80, 29.81)	−57.09%	32.78 (26.68, 40.72)	17.27 (14.12, 21.19)	−47.32%
Low-middle SDI	119.38 (57.73, 187.96)	46.50 (23.59, 68.41)	−61.05%	84.37 (58.20, 116.10)	38.76 (30.68, 48.64)	−54.06%
Low SDI	83.27 (39.14, 132.04)	40.28 (19.21, 62.10)	−51.63%	107.40 (79.20, 142.81)	55.42 (43.79, 71.93)	−48.40%

*Abbreviations:* *DALYs* Disability-adjusted life years, *SDI* Socio-demographic index

### Global burden of asthma in 2019

**Fig. 1:**
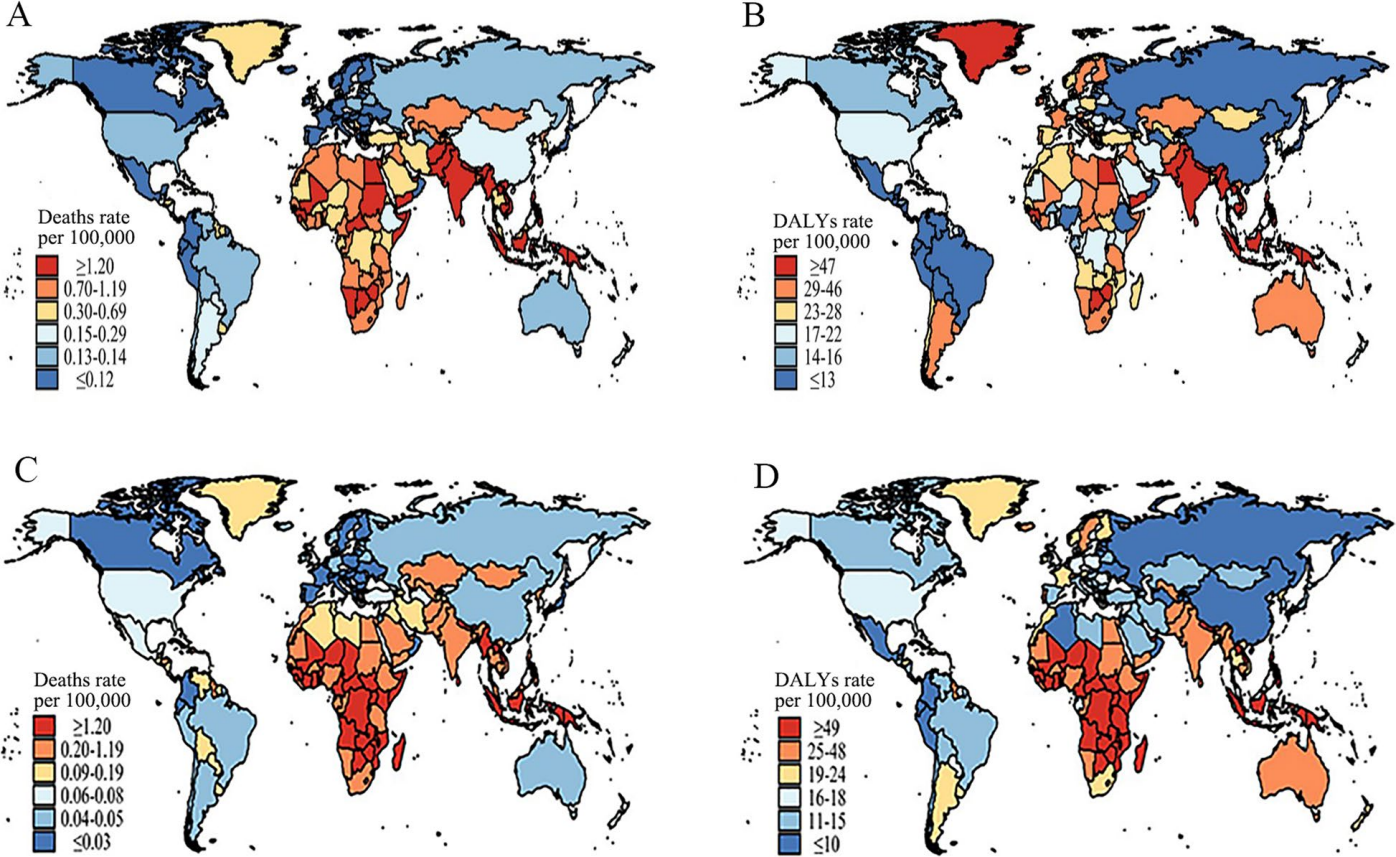
Age-standardized deaths and DALYs rates (per 100,000 population) of asthma attributed to different risk factors in 2019 stratified by country. **A** Age-standardized asthma deaths rates attributed to smoking; **B** Age-standardized asthma DALYs rates attributed to smoking; **C** Age-standardized asthma deaths rates attributed to occupational asthmagens; **D** Age-standardized asthma DALYs rates attributed to occupational asthmagens

As shown in Fig. 1, risk factors-related asthma deaths and DALYs rates had spatial heterogeneity. The top 2 sextiles of asthma deaths rate attributable to smoking (≥ 0.70 cases per 100,000 population) were seen in North and South Africa, South and Southeast Asia, Kazakhstan in Central Asia, and Mongolia in East Asia. The last two sextiles of asthma deaths rate attributable to smoking were discovered in Europe, Oceania and most American countries (Fig. 1A). Locations with ≥ 47 per 100,000 of DALYs attributable to smoking included South and Southeast Asia, Botswana, Egypt, Greenland, and Zimbabwe. With the exception of some parts of West and Central Africa, most countries in Africa and Europe located in the 2nd and 3rd sextiles (23–46 per 100,000). Countries with smoking-related asthma DALYs rate ≤ 13 per 100,000 were China, Russia and some countries in Latin America (Fig. 1B).

The top 2 sextiles (≥ 0.20 cases per 100,000 population) of asthma deaths rates attributable to occupational asthmagens were seen in almost all of Africa, South and Southeast Asia, Kazakhstan, Mongolia, and North Korea. Most of Europe, Oceania and South America, as well as Canada and China, were at the last two sextiles (≤0.05 cases per 100,000 population, Fig. 1C). Locations in top sextiles of occupational asthmagens-related asthma DALYs rates (≥ 49 per 100,000 population) included Southeast Asian countries and Sub-Saharan Africa. In Afghanistan, Australia, India, Pakistan, Uzbekistan, parts of Africa, and some European countries (such as Iceland, Ireland, Oceania and Sweden), DALYs rates attributable to occupational asthmagens were 25–48 per 100,000. Most countries in East and West Asia and America, and some countries in North Africa had low levels of DALYs rates attributable to occupational asthmagens (≤15 per 100,000 population, Fig. 1D). The asthma burden attributable to smoking and occupational asthmagens was severer in male than that in female. In contrast to male, female with top 2 sextiles of asthma DALYs rate attributable to smoking were from Argentina, Australia, Europe, Greenland and the United States (Figures S1-S2).

**Fig. 2:**
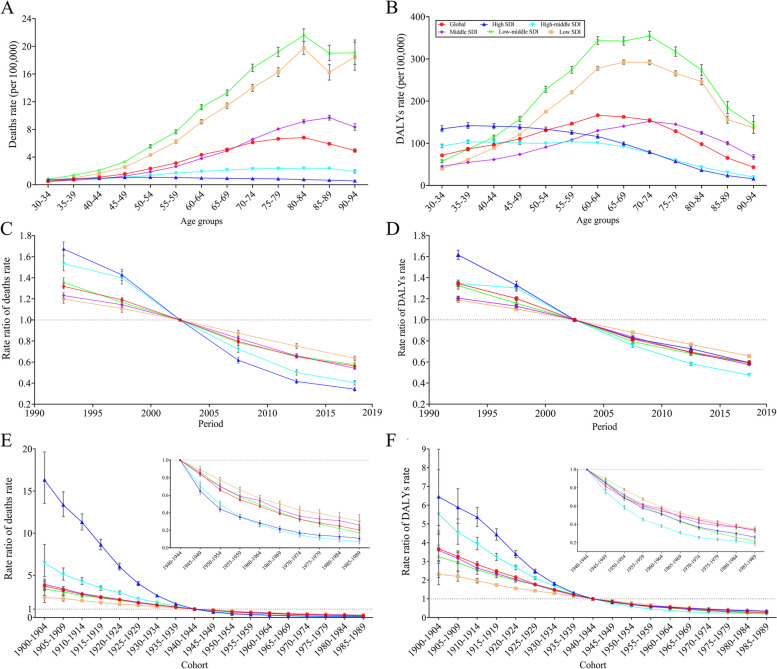
Age, period, and cohort effects on asthma deaths and DALYs rates attributed to smoking. **A** Age-specific asthma deaths rates attributed to smoking; **B** Age-specific asthma DALYs rates attributed to smoking; **C** Period rate ratio (RR) of asthma deaths rates attributed to smoking; **D** Period RR of asthma DALYs rates attributed to smoking; **E** Cohort RR of asthma deaths rates attributed to smoking; **F** Cohort RR of asthma DALYs rates attributed to smoking

### APC analysis for smoking-related asthma burden

 Global net drifts in asthma deaths and DALYs rates attributable to smoking were −3.54% [95% *confidence interval* (*CI*) = (−3.61%, −3.47%)] and −3.34% [95% *CI* = (−3.40%, −3.28%)], respectively. And local drifts of asthma deaths and DALYs rates were below −2.60% for all age groups (Table S10). Low-middle SDI region had the highest asthma deaths rate attributable to smoking across all the SDI groups, deaths rate linearly increased from 30–34 to 80–84 age groups (from 0.80 to 21.60 per 100,000), slightly decreased and then kept stable. The second highest asthma deaths rate attributable to smoking was observed in the low SDI region and the pattern change was similar to that in the low-middle SDI region. In the middle SDI region, smoking-related asthma deaths rate increased from 30–34 to 85–89 age groups, then decreased, and the rate was higher than the global level in people over 70 years. In comparison, the increase of asthma deaths rate attributable to smoking in the high and high-middle SDI regions was relatively moderate (Table 1; Fig. 2A). In each age group, asthma deaths rate attributed to smoking was higher in male than those in female (Figures S3A and S4A). There were nearly the inverse “V” shaped trends of asthma DALYs rates attributable to smoking in the low-middle, low, and middle SDI regions, and the highest values were observed in the 70–74, 65–69, and 70–74 age groups, respectively (354.62, 292.60, and 151.93 per 100,000, respectively). Asthma DALYs rate attributable to smoking in the high-SDI group was higher in the 30–44 age groups and showed a monotonic downward trend after the 35–39 age group. Similarly, this pattern occurred in the high-middle SDI region (Fig. 2B). Trends in different SDI subgroups for males were similar to the whole population, but elder female from the low SDI had the highest smoking-related asthma DALYs rate (Figures S3B and S4B). Global Asthma deaths and DALYs rates attributable to smoking decreased during 1990–2019 (Figs. 2C and D, S3C-S3D, and S4C-S4D), period RR decreasing from 1.32 [95% *CI* = (1.30, 1.34)] to 0.56 [95% *CI* = (0.55, 0.57)] and 1.34 [95% *CI* = (1.32, 1.36)] to 0.59 [95% *CI* = (0.58, 0.60)], respectively. The most significant improvements were both in the high-SDI region, with period RR decreasing from 1.67 [95% *CI* = (1.61, 1.74)] to 0.34 [95% *CI* = (0.33, 0.36)] and 1.61 [95% *CI* = (1.57, 1.66)] to 0.59 [95% *CI* = (0.57, 0.61)], respectively. Inversely, low-SDI region had the least improvement, and period RR of deaths and DALYs rate declined from 1.20 [95% *CI* = (1.16, 1.24)] to 0.64 [95% *CI* = (0.61, 0.66)] and 1.19 [95% *CI* = (1.17, 1.21)] to 0.66 [95% *CI* = (0.64, 0.67)], respectively (Fig. 2C and D). Additionally, the most conspicuous improvement in asthma deaths rate was observed in males from high-SDI regions during study period (Figure S3C). There was a downward trend for cohort effect on smoking-related asthma deaths risk. High SDI region had the highest RR from 1900–1904 to 1935–1939 birth cohorts, but ranked near bottom after the 1945–1949 cohort. In contrast, low SDI countries had the lowest RR before the 1925–1929 cohort, but ranked highest after the 1950–1954 cohort (Fig. 2E). Asthma DALYs rate attributable to smoking had a similar pattern with asthma deaths rate across the studied birth cohorts (Fig. 2F). Sex-specific RR of asthma deaths and DALYs also monotonically decreased from early to recent birth cohort, especially for males from high SDI region (Figures S3E-S3F and S4E-S4F).

### APC analysis for occupational asthmagens-related asthma burden

**Fig. 3:**
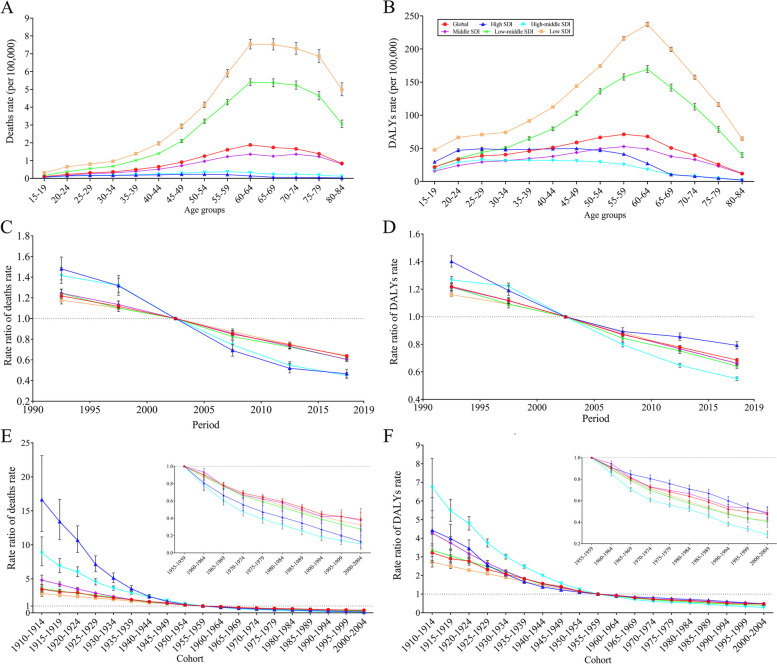
Age, period, and cohort effects on asthma deaths and DALYs rates attributed to occupational asthmagens. **A** Age-specific asthma deaths rates attributed to occupational asthmagens; **B** Age-specific asthma DALYs rates attributed to occupational asthmagens; **C** Period rate ratio (RR) of asthma deaths rates attributed to asthmagens; **D** Period RR of asthma DALYs rates attributed to occupational asthmagens; **E** Cohort RR of asthma deaths rates attributed to occupational asthmagens; **F** Cohort RR of asthma DALYs rates attributed to occupational asthmagens

 Global net drifts in asthma deaths and DALYs rates attributable to occupational asthmagens were −2.60% [95% *CI* = (−2.68%, −2.53%)] and −2.31% [95% *CI* = (−2.37%, −2.26%)], respectively. And local drifts were below −1.53% for all age groups (Table S11). Low SDI region, with the growing tendency from 15–19 to 60–64 age group (0.32 to 7.53 cases per 100,000) and then decreasing. Low SDI region had the highest asthma deaths rate attributable to occupational asthmagens, followed by the low-middle SDI region. Besides, the rate of the remaining regions was below the global level. In the middle SDI region, occupational asthmagens-related deaths rate was higher in 50–79 age groups than other age groups, with deaths rates fluctuating around 1.25. In contrast, asthma deaths rates in the high and high-middle SDI regions were far below other regions and remained flat (Fig. 3A). The trends of males and females were broadly similar to that of the general population. The male adults from low and low-middle SDI regions had higher asthma deaths rates attributable to occupational asthmagens than female adults from the same region (Figures S5A and S6A). There were the nearly inverse “V” shaped trends of asthma DALYs rates attributable to occupational asthmagens in the low, low-middle and middle SDI regions. In the low and low-middle regions, the highest values were both observed in the 60–64 age group, and the peak of middle SDI regions occurred in the 55–59 age group (52.70 per 100,000, Fig. 3B). The trends were roughly the same for both sexes and the males had higher asthma DALYs rates due to occupational asthmagens (Figures S5B and S6B). In all SDI regions, the risks of deaths and DALYs rates attributable to occupational asthmagens decreased over time, and period risk was similarly controlled for both sexes (Fig. 3C and D, S5C-S5D, and S6C-S6D). From 1990 to 2019, the period RR of asthma death rate owing to occupational asthmagens reduced from 1.48 [95% *CI* = (1.37, 1.59)] to 0.47 [95% *CI* = (0.43, 0.51)] in the high-SDI region. In the high-middle SDI region, period RR declined from 1.27 [95% *CI* = (1.24, 1.29)] to 0.55 [95% *CI* = (0.54, 0.56)]. On the contrary, the improvement in asthmagens-related asthma deaths rate was much smaller in low SDI region, with period RR of deaths rate decreasing from 1.18 [95% *CI* = (1.14, 1.22)] to 0.63 [95% *CI* = (0.61, 0.65)] and RR of DALYs rate decreasing from 1.16 [95% *CI* = (1.15, 1.17)] to 0.66 [95% *CI* = (0.66, 0.67)] (Fig. 3C and D). Cohort RR decreased monotonically in all SDI regions (Fig. 3E and F, S5E-S5F, and S6E-S6F). Similarly, the progress of cohort RR in high SDI region was more significant than low SDI region (Fig. 3E). The RR of asthma DALYs rate in the high-middle SDI region had the highest decline, from 6.73 [95% *CI* = (5.48, 8.28)] in 1910−1914 birth cohort to 0.28 [95% *CI* = (0.25, 0.32)] in 2000–2004 birth cohort (Fig. 3F). The pattern of period and cohort RRs of asthma deaths and DALYs in males and females was similar to that of the global population, but cohort RR declined more in males than females (Figures S5C-S5F and S6C-S6F).

## Discussion

This was the first study to perform APC analysis of risk factors-specific (smoking and occupational asthmagens) asthma deaths and DALYs rates during 1990–2019 at global and regional levels. There were striking improvements in smoking- and occupational asthmagens-related asthma burden worldwide. Results from APC analysis showed that asthma deaths and DALYs rates attributable to smoking and occupational asthmagens sharply increased in the middle-aged adults and peak value occurred in the elders. Importantly, low and low-middle SDI regions had higher asthma deaths and DALYs rates than other regions in nearly all age groups. For the period and cohort RRs of smoking-related asthma burden, the most significant improvement occurred in high SDI region. What’s more, high SDI region also showed the most significant improvement of asthma deaths rate attributable to occupational asthmagens. High-middle SDI regions showed more improvements in RRs of asthma DALYs rates. Low SDI region showed least progress in period and cohort RRs than other regions. Besides, there was a sex disparity in asthma burden, male had higher asthma burden than female despite their similar change pattern. Our findings showed that tobacco control and occupational protection could help reduce asthma burden. Policymakers from lower SDI regions should reinforce tobacco control and prioritize workplace protection.

Tobacco smoke is an essential risk factor for asthma burden, and smoking-related asthma burden dropped by more than half in the past three decades. Tobacco smoke could deteriorate inflammation, aggravate respiratory virus infections, induce airway changes, and skew immune responses [19]. In 2019, asthma burden attributable to smoking was high in Southeast Asia and Africa, but the underlying reasons were different. Southeast Asia was one of the world’s most extensive tobacco epidemic areas, and almost 50% of males and 40% of females consumed some forms of tobacco [20]. High smoking rates put them stuck at an enhancive risk of severe asthma symptoms and minor response to steroid therapy [21]. But in Africa, the increase in smoking rates and nicotine exposure, lack of trained medical staff and diagnostic apparatus, and inaccessible and unaffordable inhaled medications contributed to high smoking-related asthma deaths and DALYs rates [8, 22]. Sex disparities in asthma burden attributable to smoking were associated with smoking rate. Females from Europe and Oceania had the highest prevalence of smoking [23]. According to our results from APC analysis, the asthma deaths rate increased with age and peaked in elders, which was similar to the previous studies [24]. Most smokers had a stable smoking pattern until later life [25], and underwent progressive and cumulative lung damage [26]. Together with aging-induced decline in immunity and lung function [27], elderly smokers were more likely to develop asthma and die. In all age groups, smoking-related asthma deaths risks in the middle, low-middle, and low SDI regions showed a similar pattern, surpassing other SDI groups significantly. Compared with other regions, smoking intensity was lower and smoking quit rate was higher in the high and high-middle SDI regions [23, 28]. Mattiuzzi et al. showed that asthma DALYs attributed to smoking reached their peak between 55 and 70 years old in both males and females in the worldwide [29]. With more detailed age groups, we found the highest smoking-related asthma DALYs rate in the 60–64 age group in both sexes. Asthma DALYs attributed to smoking in low and low-middle SDI regions were mainly from YLLs, but YLDs accounted for a substantial part of DALYs in high and high-middle SDI regions. Asthma DALYs rate attributed to smoking was higher in younger populations from high SDI region than other regions, like Europe and the Americas, which might be associated with an earlier age of smoking initiation [30]. The substantial decline in period RR of smoking-related asthma burden may be related to global smoking prevalence declined since 1990, particularly after the passage of the *World Health Organization Framework Convention on Tobacco Control* in 2005 [31]. Compared with large declines in the Americas and Europe, the Africa and Southeast Asia showed a net increase in male smokers since 2000 [32]. According to the report of World Health Organization on the global tobacco epidemic in 2019, 49 of 59 countries that have not taken any tobacco control strategies were low-income and middle-income countries, which may lead to less decrease in period and cohort RRs in low-SDI group [33]. Robust tobacco control measures benefited the new birth cohort with a better environment and resulted in lower smoking-related asthma burden. In the future, it is imperative to fortify measures aimed at tobacco control, proactively thwart the initiation of smoking among adolescents, and improve accessibility to inhaled medications, especially for people in Africa.

Occupational asthmagens are also important risk factor for asthma. A survey of 13 countries reported that about 10-25% of adult asthma was attributed to occupational factors [34]. In developing countries, such as Africa and South Asia, occupational asthma was the second most common occupational lung disease [35], and both occupational asthmagens-related asthma deaths and DALYs rates were high. In contrast to developed countries, developing countries did not vigorously monitor the workplace exposure or set strict exposure limits. In addition, due to constraint of medical resources, healthcare providers could not recognize the key signs and give definite diagnosis in the early stage of occupational asthma development. What’s worse, some clinicians did not ask about any potential occupational hazards [36]. Furthermore, our results indicated occupational asthmagens-related asthma DALYs rates were higher in Australia, Iceland and Sweden than other developed countries. A review reported Scandinavian countries had higher incidence of occupational asthma than Western Europe, United States of America, South Africa, and Brazil [35]. Diisocyanate was the leading cause of occupational asthma in industrialized countries [37], and diisocyanate-related new cases of occupational asthma was estimated to exceed 5,000 per year in the European Union [38]. We showed that people aged 60–64 years old had the highest asthma deaths and DALYs rate attributed to occupational asthmagens, which was similar with the previous studies [39]. Asthma burden remained high after retirement, suggesting cumulative and lagged effects. After repeating exposure to low levels of asthmagens (occupations like professional cleaners, pastry chefs, and oil sprayers), asthma developed insidiously over time [40]. Worse still was even if occupational asthma patients were removed from exposure, respiratory symptoms persisted and aggravated [41]. For occupational asthma deaths and DALYs rates, our findings demonstrated that the period and cohort RRs showed a declining trend in all regions. Promotion of respiratory protection in industry, introduction of sensitive diagnostic methods, and implementation of occupational avoidance could reduce the burden of occupational asthma [37]. In 1970, the Occupational Safety and Health Administration was introduced in the USA, and it was the first occupational regulation. In 1978, the European Community conducted harmonizing measures to protect workers’ health, and adopted a legal framework for workplace chemicals in 1980, setting a series of indicative exposure limits [42]. These policies have also obtained some achievements, for example, in France, the number of cases compensated for occupational asthma fell from 142 in 1991 to 23 in 2016 [37]. Besides, Australian Aluminium Council held regular health panel meetings since 1990, educated employees about occupational asthma, and conducted regular health examination to reduce asthma burden [43]. However, until 2010, Asian organizations did not have harmonized regulations on occupational exposure limit settings or workplace protection [37, 43, 44]. All these may explain why period and cohort RRs decreased more in the high and middle-high SDI regions than other regions. With the rapid development of industrialization in low SDI regions, the related occupational risk factors should be addressed. Creating a good working environment where the exposure value is below the defined hazard range, and early recognition of workers with occupational symptoms may reduce the burden of occupational asthma. Although burden of occupational asthma continued to decrease, there are also massive opportunities to achieve conspicuous improvement by maintaining and reinforcing control of asthmagens. There may be many neglected agents with strong stimulatory effects on the airway, and comprehensive recording and monitoring of traditional and novel irritants in the workplace are necessary.

The present study had several advantages. Firstly, we provided new insights into the global patterns of asthma deaths and DALYs rates related to environmental risk factors, which would benefit public health policy making and prevent asthma development in a precise manner. Secondly, we analyzed time trends of asthma burden in the five SDI regions, and our findings would help to demonstrate regional disparity and difference in the decrease of asthma deaths and DALYs rates. Thirdly, the APC model was applied to decompose age, period, and cohort effects on asthma burden. Moreover, net and local drifts were generated to evaluate overall and age-specific annual percent changes. However, there were some limitations. Firstly, this study only carried out a descriptive analysis of GBD 2019 data and only two environmental risk factors were available. Other risk factors for asthma, such as air pollutants, environmental allergens, and respiratory viruses, were not included. Secondly, in the GBD 2019, definition of asthma was based on clinical assessment and/or self-report [16], so it is probably influenced by recall bias, acquisition of health services, and different origins of survey questions. Thirdly, because of the fixed and equal age and period intervals required by APC tool, data of people aged ≥ 95 years for smoking-related asthma deaths and DALYs rates were not included in the analyses, as well as data of people aged ≥ 85 years for occupational asthmagens-related asthma burden. And APC analyses for the smoking-related asthma burden of 0–29 age group and occupational asthmagens-related asthma burden of 0–14 age group were not performed due to data availability.

## Conclusion

Smoking- and occupational asthmagens-related asthma burden dropped by more than 45% during 1990–2019. In 2019, there were region and sex disparities in environmental factors-related asthma burden, and higher burden was observed in Africa, South and Southeast Asia, as well as male. Peaks of smoking- and occupational asthmagens-related asthma burden occurred in the elders, suggesting cumulative and lagged effects of environmental exposures. The most conspicuous improvements in period and cohort effects on asthma burden appeared in regions with higher SDI indexes, and least improvements in low SDI region. Decision-makers in low SDI region should strengthen tobacco control and prioritize workplace protection.

### Supplementary Information

**Additional file 1:** **Table S1.** Quintiles of different SDI groups. **Table S2.** Countries and territories of different SDI groups in 1990 and 2019. **Table S3.** Full list of selected covariates for the CODEm models in the asthma estimation. **Table S4.** Lay description, disability weight and proportion of different levels of asthma severity. **Table S5.** 22 occupational asthmagens recorded in the International Labor Organization. **Table S6.** Change in age-standardized asthma deaths number related to different risk factors from 1990 to 2019. **Table S7.** Change in age-standardized asthma deaths percent related to different risk factors from 1990 to 2019. **Table S8.** Change in age-standardized asthma DALYs number related to different risk factors from 1990 to 2019. **Table S9.** Change in age-standardized asthma DALYs percent related to different risk factors from 1990 to 2019. **Table S10.** Net and local drifts for asthma deaths and DALYs rates attributed to smoking (%). **Table S11.** Net and local drifts for asthma deaths and DALYs rates attributed to occupational asthmagens (%). **Figure S1.** Age-standardized male asthma deaths and DALYs rates attributed to different risk factors in 2019 by countries. **Figure S2.** Age-standardized female asthma deaths and DALYs rates attributed to different risk factors in 2019 by countries. **Figure S3.** Age, period, and cohort effects on asthma deaths and DALYs rates attributed to smoking for male. **Figure S4.** Age, period, and cohort effects on asthma deaths and DALYs rates attributed to smoking for female. **Figure S5.** Age, period, and cohort effects on asthma deaths and DALYs rates attributed to occupational asthmagens for male. **Figure S6.** Age, period, and cohort effects on asthma deaths and DALYs rates attributed to occupational asthmagens for female.

## Data Availability

All data could be extracted from the online GBD repository, https://vizhub.healthdata.org/gbd-results.

## Abbreviations

APC: Age-period-cohort
CI: Confidence interval
CODEm: Combined Cause of Death Model
DALYs: Disability-adjusted life years
GBD: Global Burden of Disease
RR: Rate ratio
SDI: Socio-demographic index
UI: Uncertainty interval
YLLs: Years of life lost
