# Supplementary material for: Global patterns of asthma burden related to environmental risk factors during 1990–2019: an age-period-cohort analysis for global burden of disease study 2019
Source: Environ Health. 2024 Feb 15;23:20. doi: 10.1186/s12940-024-01060-8 (PMC10868053; doi:10.1186/s12940-024-01060-8)

**Supplemental material**

**Global patterns of asthma burden related to environmental risk factors during 1990-2019: An age-period-cohort analysis for Global Burden of Disease Study 2019**

Siying Zhang, Zongshi Gao, Lihong Wu, Yumei Zhong, Hui Gao, Fang-biao Tao, Xiulong Wu

**Table of Contents**

**Table S1:** Quintiles of different SDI groups.

**Table S2:** Countries and territories of different SDI groups in 1990 and 2019.

**Table S3:** Full list of selected covariates for the CODEm models in the asthma estimation.

**Table S4:** Lay description, disability weight and proportion of different levels of asthma severity.

**Table S5:** 22 occupational asthmagens recorded in the International Labor Organization.

**Table S6:** Change in age-standardized asthma deaths number related to different risk factors from 1990 to 2019.

**Table S7:** Change in age-standardized asthma deaths percent related to different risk factors from 1990 to 2019.

**Table S8:** Change in age-standardized asthma DALYs number related to different risk factors from 1990 to 2019.

**Table S9:** Change in age-standardized asthma DALYs percent related to different risk factors from 1990 to 2019.

**Table S10:** Net and local drifts for asthma deaths and DALYs rates attributed to smoking (%).

**Table S11:** Net and local drifts for asthma deaths and DALYs rates attributed to occupational asthmagens (%).

**Figure S1:** Age-standardized male asthma deaths and DALYs rates attributed to different risk factors in 2019 by countries.

**Figure S2:** Age-standardized female asthma deaths and DALYs rates attributed to different risk factors in 2019 by countries.

**Figure S3:** Age, period, and cohort effects on asthma deaths and DALYs rates attributed to smoking for male.

**Figure S4:** Age, period, and cohort effects on asthma deaths and DALYs rates attributed to smoking for female.

**Figure S5:** Age, period, and cohort effects on asthma deaths and DALYs rates attributed to occupational asthmagens for male.

**Figure S6:** Age, period, and cohort effects on asthma deaths and DALYs rates attributed to occupational asthmagens for female.

| **Table S1:** Quintiles of different SDI groups. | | |
| --- | --- | --- |
| SDI quintile | lower bound | upper bound |
| Low SDI | 0 | 0.454743 |
| Low-middle SDI | 0.454743 | 0.607679 |
| Middle SDI | 0.607679 | 0.689504 |
| High-middle SDI | 0.689504 | 0.805129 |
| High SDI | 0.805129 | 1 |
| **Notes:** SDI, socio-demographic index; each SDI group contained an upper bound but no lower bound when estimating. | | |

| **Table S2:** Countries and territories of different SDI groups in 1990 and 2019. | | |
| --- | --- | --- |
| **Countries and territories** | **1990** | **2019** |
| Afghanistan | Low SDI | Low SDI |
| Albania | Low-middle SDI | Middle SDI |
| Algeria | Low SDI | Middle SDI |
| American Samoa | Low-middle SDI | High-middle SDI |
| Andorra | High SDI | High SDI |
| Angola | Low SDI | Low-middle SDI |
| Antigua and Barbuda | Low-middle SDI | High-middle SDI |
| Argentina | Low-middle SDI | High-middle SDI |
| Armenia | Low-middle SDI | Middle SDI |
| Australia | High-middle SDI | High SDI |
| Austria | High-middle SDI | High SDI |
| Azerbaijan | Low-middle SDI | Middle SDI |
| Bahamas | High-middle SDI | High-middle SDI |
| Bahrain | Low SDI | High-middle SDI |
| Bangladesh | Middle SDI | Low-middle SDI |
| Barbados | Low SDI | High-middle SDI |
| Belarus | Low SDI | High-middle SDI |
| Belgium | Middle SDI | High SDI |
| Belize | Low SDI | Low-middle SDI |
| Benin | Low SDI | Low SDI |
| Bermuda | Low SDI | High SDI |
| Bhutan | Low SDI | Low-middle SDI |
| Bolivia (Plurinational State of) | Low SDI | Low-middle SDI |
| Bosnia and Herzegovina | Low SDI | High-middle SDI |
| Botswana | Middle SDI | Middle SDI |
| Brazil | Low-middle SDI | Middle SDI |
| Brunei Darussalam | High-middle SDI | High SDI |
| Bulgaria | Low SDI | High-middle SDI |
| Burkina Faso | High-middle SDI | Low SDI |
| Burundi | Low SDI | Low SDI |
| Cabo Verde | Middle SDI | Low-middle SDI |
| Cambodia | High-middle SDI | Low-middle SDI |
| Cameroon | Low-middle SDI | Low-middle SDI |
| Canada | Low SDI | High SDI |
| **Countries and territories** | **1990** | **2019** |
| Central African Republic | Low SDI | Low SDI |
| Chad | Low SDI | Low SDI |
| Chile | Low-middle SDI | High-middle SDI |
| China | Low SDI | Middle SDI |
| Colombia | Low-middle SDI | Middle SDI |
| Comoros | Low SDI | Low SDI |
| Congo | Low SDI | Low-middle SDI |
| Cook Islands | Middle SDI | High-middle SDI |
| Costa Rica | Low-middle SDI | Middle SDI |
| Côte d'Ivoire | Low SDI | Low SDI |
| Croatia | Middle SDI | High-middle SDI |
| Cuba | Low-middle SDI | Middle SDI |
| Cyprus | Middle SDI | High SDI |
| Czechia | Middle SDI | High SDI |
| Democratic People's Republic of Korea | Low SDI | Low-middle SDI |
| Democratic Republic of the Congo | Low SDI | Low SDI |
| Denmark | High SDI | High SDI |
| Djibouti | Low SDI | Low-middle SDI |
| Dominica | Low-middle SDI | High-middle SDI |
| Dominican Republic | Low SDI | Low-middle SDI |
| Ecuador | Low-middle SDI | Middle SDI |
| Egypt | Low SDI | Middle SDI |
| El Salvador | Low SDI | Low-middle SDI |
| Equatorial Guinea | Low SDI | Middle SDI |
| Eritrea | Low SDI | Low SDI |
| Estonia | Middle SDI | High SDI |
| Eswatini | Low SDI | Low-middle SDI |
| Ethiopia | Low SDI | Low SDI |
| Fiji | Low-middle SDI | Middle SDI |
| Finland | High-middle SDI | High SDI |
| France | High-middle SDI | High SDI |
| Gabon | Low SDI | Middle SDI |
| Gambia | Low SDI | Low SDI |
| Georgia | Middle SDI | High-middle SDI |
| Germany | High SDI | High SDI |
| **Countries and territories** | **1990** | **2019** |
| Ghana | Low SDI | Low-middle SDI |
| Greece | Middle SDI | High-middle SDI |
| Greenland | Middle SDI | High-middle SDI |
| Grenada | Low-middle SDI | Middle SDI |
| Guam | High-middle SDI | High SDI |
| Guatemala | Low SDI | Low-middle SDI |
| Guinea | Low SDI | Low SDI |
| Guinea-Bissau | Low SDI | Low SDI |
| Guyana | Low SDI | Middle SDI |
| Haiti | Low SDI | Low SDI |
| Honduras | Low SDI | Low-middle SDI |
| Hungary | Middle SDI | High-middle SDI |
| Iceland | High-middle SDI | High SDI |
| India | Low SDI | Low-middle SDI |
| Indonesia | Low SDI | Middle SDI |
| Iran (Islamic Republic of) | Low SDI | Middle SDI |
| Iraq | Low SDI | Middle SDI |
| Ireland | High-middle SDI | High SDI |
| Israel | High-middle SDI | High-middle SDI |
| Italy | High-middle SDI | High-middle SDI |
| Jamaica | Low-middle SDI | Middle SDI |
| Japan | High-middle SDI | High SDI |
| Jordan | Low-middle SDI | High-middle SDI |
| Kazakhstan | Low-middle SDI | High-middle SDI |
| Kenya | Low SDI | Low-middle SDI |
| Kiribati | Low SDI | Low-middle SDI |
| Kuwait | Middle SDI | High SDI |
| Kyrgyzstan | Low-middle SDI | Low-middle SDI |
| Lao People's Democratic Republic | Low SDI | Low-middle SDI |
| Latvia | Middle SDI | High SDI |
| Lebanon | Low-middle SDI | High-middle SDI |
| Lesotho | Low SDI | Low-middle SDI |
| Liberia | Low SDI | Low SDI |
| Libya | Low SDI | High-middle SDI |
| Lithuania | Middle SDI | High SDI |
| **Countries and territories** | **1990** | **2019** |
| Luxembourg | High SDI | High SDI |
| Madagascar | Low SDI | Low SDI |
| Malawi | Low SDI | Low SDI |
| Malaysia | Low-middle SDI | High-middle SDI |
| Maldives | Low SDI | Low-middle SDI |
| Mali | Low SDI | Low SDI |
| Malta | Middle SDI | High-middle SDI |
| Marshall Islands | Low SDI | Low-middle SDI |
| Mauritania | Low SDI | Low-middle SDI |
| Mauritius | Low-middle SDI | High-middle SDI |
| Mexico | Low-middle SDI | Middle SDI |
| Micronesia (Federated States of) | Low SDI | Low-middle SDI |
| Monaco | High SDI | High SDI |
| Mongolia | Low-middle SDI | Low-middle SDI |
| Montenegro | High-middle SDI | High-middle SDI |
| Morocco | Low SDI | Low-middle SDI |
| Mozambique | Low SDI | Low SDI |
| Myanmar | Low SDI | Low-middle SDI |
| Namibia | Low SDI | Middle SDI |
| Nauru | Low-middle SDI | Middle SDI |
| Nepal | Low SDI | Low SDI |
| Netherlands | High-middle SDI | High SDI |
| New Zealand | High-middle SDI | High SDI |
| Nicaragua | Low SDI | Low-middle SDI |
| Niger | Low SDI | Low SDI |
| Nigeria | Low SDI | Low-middle SDI |
| Niue | Low-middle SDI | High-middle SDI |
| North Macedonia | Middle SDI | High-middle SDI |
| Northern Mariana Islands | High-middle SDI | High-middle SDI |
| Norway | High SDI | High SDI |
| Oman | Low SDI | High-middle SDI |
| Pakistan | Low SDI | Low SDI |
| Palau | Middle SDI | High-middle SDI |
| Palestine | Low SDI | Low-middle SDI |
| Panama | Low-middle SDI | Middle SDI |
| **Countries and territories** | **1990** | **2019** |
| Papua New Guinea | Low SDI | Low SDI |
| Paraguay | Low-middle SDI | Middle SDI |
| Peru | Low-middle SDI | Middle SDI |
| Philippines | Low-middle SDI | Middle SDI |
| Poland | Middle SDI | High-middle SDI |
| Portugal | Low-middle SDI | High-middle SDI |
| Puerto Rico | Middle SDI | High SDI |
| Qatar | Low-middle SDI | High SDI |
| Republic of Korea | Middle SDI | High SDI |
| Republic of Moldova | Low-middle SDI | High-middle SDI |
| Romania | Middle SDI | High-middle SDI |
| Russian Federation | High-middle SDI | High-middle SDI |
| Rwanda | Low SDI | Low SDI |
| Saint Kitts and Nevis | Low-middle SDI | High-middle SDI |
| Saint Lucia | Low-middle SDI | Middle SDI |
| Saint Vincent and the Grenadines | Low-middle SDI | Middle SDI |
| Samoa | Low-middle SDI | Middle SDI |
| San Marino | High SDI | High SDI |
| São Tomé and Príncipe | Low SDI | Low-middle SDI |
| Saudi Arabia | Low-middle SDI | High SDI |
| Senegal | Low SDI | Low SDI |
| Serbia | Middle SDI | High-middle SDI |
| Seychelles | Low-middle SDI | High-middle SDI |
| Sierra Leone | Low SDI | Low SDI |
| Singapore | Middle SDI | High SDI |
| Slovakia | Middle SDI | High SDI |
| Slovenia | High-middle SDI | High SDI |
| Solomon Islands | Low SDI | Low SDI |
| Somalia | Low SDI | Low SDI |
| South Africa | Low-middle SDI | Middle SDI |
| South Sudan | Low SDI | Low SDI |
| Spain | Middle SDI | High-middle SDI |
| Sri Lanka | Low-middle SDI | High-middle SDI |
| Sudan | Low SDI | Low-middle SDI |
| Suriname | Low-middle SDI | Middle SDI |
| **Countries and territories** | **1990** | **2019** |
| Sweden | High-middle SDI | High SDI |
| Switzerland | High SDI | High SDI |
| Syrian Arab Republic | Low SDI | Middle SDI |
| Taiwan (Province of China) | Middle SDI | High SDI |
| Tajikistan | Low-middle SDI | Low-middle SDI |
| Thailand | Low-middle SDI | Middle SDI |
| Timor-Leste | Low SDI | Low-middle SDI |
| Togo | Low SDI | Low SDI |
| Tokelau | Low SDI | Middle SDI |
| Tonga | Low-middle SDI | Middle SDI |
| Trinidad and Tobago | Middle SDI | High-middle SDI |
| Tunisia | Low SDI | Middle SDI |
| Turkey | Low-middle SDI | High-middle SDI |
| Turkmenistan | Low-middle SDI | Middle SDI |
| Tuvalu | Low SDI | Low-middle SDI |
| Uganda | Low SDI | Low SDI |
| Ukraine | Middle SDI | High-middle SDI |
| United Arab Emirates | Middle SDI | High SDI |
| United Kingdom | High-middle SDI | High SDI |
| United Republic of Tanzania | Low SDI | Low SDI |
| United States of America | High-middle SDI | High SDI |
| United States Virgin Islands | Middle SDI | High-middle SDI |
| Uruguay | Low-middle SDI | High-middle SDI |
| Uzbekistan | Low-middle SDI | Middle SDI |
| Vanuatu | Low SDI | Low-middle SDI |
| Venezuela (Bolivarian Republic of) | Low-middle SDI | Low-middle SDI |
| Viet Nam | Low SDI | Middle SDI |
| Yemen | Low SDI | Low SDI |
| Zambia | Low SDI | Low-middle SDI |
| Zimbabwe | Low SDI | Low-middle SDI |
| **Abbreviation:** SDI, socio-demographic index. | | |

| **Table S3:** Full list of selected covariates for the CODEm models in the asthma estimation [1]. | | |
| --- | --- | --- |
| Level | Covariate | Direction |
| 1 | Log-transformed SEV scalar: asthma | 1 |
|  | Cumulative cigarettes (10 years) | 1 |
|  | Cumulative cigarettes (5 years) | 1 |
|  | Healthcare access and quality index | −1 |
| 2 | Smoking prevalence | 1 |
|  | Indoor air pollution (all cooking fuels) | 1 |
|  | Outdoor air pollution (PM2.5) | 1 |
| 3 | Lagged 10 years LDI (I$ per capita) | −1 |
|  | Education (years per capita) | −1 |
|  | Socio-demographic index | −1 |
| **Notes:** "1" means that the direction of the covariate is positive, "−1" means that the direction of the covariate is negative. | | |

| **Table S4:** Lay description, disability weight and proportion of different levels of asthma severity [1]. | | | |
| --- | --- | --- | --- |
| Severity level | Lay description | Disability weight (95% CI) | Proportion |
| Asymptomatic | No symptoms | 0 | 36.2% (35.0%, 37.3%) |
| Controlled | This person has wheezing and cough once a month, which does not cause difficulty with daily activities. | 0.015 (0.007, 0.026) | 19.9% (13.6%, 27.8%) |
| Partially controlled | This person has wheezing and cough once a week, which causes some difficulty with daily activities. | 0.036 (0.022, 0.055) | 20.6% (15.1%, 25.8%) |
| Uncontrolled | This person has wheezing, cough, and shortness of breath more than twice a week, which causes difficulty with daily activities and sometimes wakes the person at night. | 0.133 (0.086, 0.192) | 23.3% (18.7%, 30.3%) |

| **Table S5:** 22 occupational asthmagens recorded in the International Labor Organization [2]. | | |
| --- | --- | --- |
| Categories | Description | |
| Low risk | Unlikely to be exposed to substances associated with risk of asthma or to other irritating chemicals | |
|  | Possible exposure to other substances (not typically associated with asthma) | Low level exposure to chemicals which may or may not be sensitisers |
|  |  | Exposure to irritants, but not high peaks (in construction, mining, etc) |
|  |  | Exposure to exhaust fumes and environmental tobacco smoke |
| High risk | Jobs with a moderate or high probability of exposure to agents associated with occupational asthma | **High molecular weight agents:** Derived from animals (rodents, livestock, fish, shellfish); arthropods or mites; derived from plants (latex, flour, other, miscellaneous); bioaerosols (moulds, endotoxins, etc); biological enzymes. |
|  |  | **Low molecular weight agents:** Highly reactive chemicals (cross linking agents) (anhydrides, amines, reactive dyes, glues, biocides, others, etc); isocyanates; sensitising drugs; industrial cleaning agents; wood dusts, sensitizing; metal sensitisers. |
|  |  | **Mixed environments or agents:** Jobs with high probability of exposure to components associated with metal working fluids; jobs in agriculture with high probability of exposure to organic particulate or fumes; textile industry production jobs; jobs with moderate to high probability of accidental or periodic exposure to very high levels of irritant gases or fumes (peak exposures). |
| Individual re-evaluation required | Imprecise exposure estimation according to the job code: requires further verification by an expert after taking into account actual job title or industry sector | |

| **Table S6:** Change in age-standardized asthma deaths number related to different risk factors from 1990 to 2019. | | | | | | | |
| --- | --- | --- | --- | --- | --- | --- | --- |
| Categories | Smoking-related asthma deaths number (thousand) | | |  | Occupational asthmagens-related asthma deaths number (thousand) | | |
|  | 1990 | 2019 | Percent |  | 1990 | 2019 | Percent |
|  |  |  | change |  |  |  | change |
| Global | 69.09 (36.27, 104.10) | 54.85 (29.15, 78.01) | −20.61% |  | 39.18 (28.16, 52.93) | 34.40 (27.83, 42.61) | −12.20% |
| Sex |  |  |  |  |  |  |  |
| Male | 57.33 (29.77, 85.84) | 44.65 (24.00, 62.28) | −22.12% |  | 27.80 (18.43,39.94) | 24.08 (18.39, 30.55) | −13.38% |
| Female | 11.76 (5.88, 19.42) | 10.19 (4.66, 16.25) | −13.35% |  | 11.38 (7.16,17.03) | 10.31 (7.50,13.50) | −9.40% |
| SDI region |  |  |  |  |  |  |  |
| High SDI | 8.06 (4.57, 11.53) | 2.54 (1.37, 3.76) | −68.49% |  | 1.87 (1.70,1.99) | 0.81 (0.74, 0.91) | −56.68% |
| High-middle SDI | 9.79 (5.54, 13.81) | 4.75 (2.66, 6.63) | −1.48% |  | 3.28 (2.79, 4.05) | 1.67 (1.44, 1.98) | −49.09% |
| Middle SDI | 17.41 (9.55, 25.73) | 16.33 (8.96, 22.64) | −6.20% |  | 8.95 (7.12, 11.76) | 8.10 (7.00,9.67) | −9.50% |
| Low-middle SDI | 26.63 (13.08, 43.15) | 23.77 (11.95, 35.59) | −10.74% |  | 16.80 (11.07, 24.08) | 15.18 (11.18, 20.18) | −9.64% |
| Low SDI | 7.16 (3.27, 11.79) | 7.41 (3.52, 11.64) | 3.49% |  | 8.26 (5.74, 11.50) | 8.61 (6.61, 11.86) | 4.24% |
| **Abbreviation:** SDI, socio-demographic index. | | | | | | | |

| **Table S7:** Change in age-standardized asthma deaths percent related to different risk factors from 1990 to 2019. | | | | | | | |
| --- | --- | --- | --- | --- | --- | --- | --- |
| Categories | Smoking-related asthma deaths percent  (per 100,000 population) | | |  | Occupational asthmagens-related asthma deaths percent  (per 100,000 population) | | |
|  | 1990 | 2019 | Percent |  | 1990 | 2019 | Percent |
|  |  |  | change |  |  |  | change |
| Global | 15.21 (8.53, 20.75) | 11.71 (6.09, 16.18) | −23.01% |  | 7.70 (6.49, 8.92) | 7.10 (6.19, 8.08) | −7.79% |
| Sex |  |  |  |  |  |  |  |
| Male | 25.01 (14.31, 33.32) | 20.49 (11.18, 28.04) | −18.07% |  | 9.95 (8.38, 11.49) | 10.03 (8.75, 11.33) | 0.80% |
| Female | 5.29 (2.68, 7.45) | 4.07 (1.92, 5.99) | −23.06% |  | 4.79 (3.91, 5.75) | 4.22 (3.44, 5.02) | −11.90% |
| SDI region |  |  |  |  |  |  |  |
| High SDI | 21.60 (12.46, 31.01) | 14.57 (7.72, 21.21) | −32.55% |  | 5.46 (5.16, 5.82) | 6.29 (5.78, 7.02) | 15.20% |
| High-middle SDI | 17.35 (9.77, 23.69) | 12.90 (7.10, 18.11) | −25.65% |  | 5.30 (4.76, 5.79) | 4.63 (4.18, 5.26) | −12.64% |
| Middle SDI | 14.73 (8.34, 19.78) | 13.31 (7.26, 18.54) | −9.64% |  | 5.89 (5.07, 6.70) | 5.75 (5.18, 6.44) | −2.38% |
| Low-middle SDI | 15.48 (8.29, 21.87) | 11.71 (5.82, 16.40) | −24.35% |  | 7.93 (6.18, 9.71) | 6.43 (5.17, 7.86) | −18.92% |
| Low SDI | 10.15 (4.96, 14.61) | 8.54 (4.17, 12.40) | −15.86% |  | 9.34 (7.46, 11.32) | 7.81 (6.40, 9.37) | −16.38% |
| **Abbreviation:** SDI, socio-demographic index. | | | | | | | |

| **Table S8:** Change in age-standardized asthma DALYs number related to different risk factors from 1990 to 2019. | | | | | | | |
| --- | --- | --- | --- | --- | --- | --- | --- |
| Categories | Smoking-related asthma DALYs number (thousand) | | |  | Occupational asthmagens-related asthma DALYs number (thousand) | | |
|  | 1990 | 2019 | Percent |  | 1990 | 2019 | Percent |
|  |  |  | change |  |  |  | change |
| Global | 2635.33 (1459.80, 3770.74) | 2122.14 (1127.41, 3006.48) | −19.47% |  | 1951.43 (1527.84, 2441.34) | 1896.34 (1510.61, 2334.59) | −2.82% |
| Sex |  |  |  |  |  |  |  |
| Male | 1999.76 (1096.29, 2837.62) | 1599.96 (871.05, 2214.17) | −19.99% |  | 1307.05 (981.28, 1688.55) | 1238.49 (975.30, 1529.54) | −5.25% |
| Female | 635.57 (319.29, 952.31) | 522.18 (258.93, 787.89) | −17.84% |  | 644.38 (478.85, 830.37) | 657.86 (511.33, 842.03) | 2.09% |
| SDI region |  |  |  |  |  |  |  |
| High SDI | 660.81 (346.57, 983.50) | 397.13 (195.69, 619.43) | −39.90% |  | 298.91 (215.31, 407.98) | 249.55 (171.93, 354.35) | −16.51% |
| High-middle SDI | 477.22 (265.75, 670.25) | 298.09 (156.42, 433.73) | −37.54% |  | 254.50 (195.57, 329.31) | 184.20 (135.88,248.42) | −27.62% |
| Middle SDI | 542.29 (304.90, 767.50) | 554.55 (304.88, 767.51) | 2.26% |  | 440.72 (359.01, 547.63) | 462.62 (378.28, 564.78) | 4.97% |
| Low-middle SDI | 743.58 (366.07, 1145.99) | 648.65 (329.93, 951.61) | −12.77% |  | 622.21 (441.58, 839.45) | 606.29 (481.47, 758.10) | −2.56% |
| Low SDI | 209.78 (100.77, 325.29) | 222.12 (105.84, 335.89) | 5.88% |  | 334.04 (251.56, 426.83) | 392.31 (312.91, 492.76) | 17.44% |
| **Abbreviations:** DALYs, disability adjusted life year; SDI, socio-demographic index. | | | | | | | |

| **Table S9:** Change in age-standardized asthma DALYs percent related to different risk factors from 1990 to 2019. | | | | | | | |
| --- | --- | --- | --- | --- | --- | --- | --- |
| Categories | Smoking-related asthma DALYs percent  (per 100,000 population) | | |  | Occupational asthmagens-related asthma DALYs percent (per 100,000 population) | | |
|  | 1990 | 2019 | Percent |  | 1990 | 2019 | Percent |
|  |  |  | change |  |  |  | change |
| Global | 13.28 (7.41, 18.13) | 9.36 (5.17, 12.77) | −29.52% |  | 8.89 (7.87, 10.02) | 8.39 (7.54, 9.28) | −5.62% |
| Sex |  |  |  |  |  |  |  |
| Male | 20.70 (12.16, 27.52) | 14.85 (8.39, 20.14) | −28.26% |  | 11.65 (10.11, 13.16) | 11.14 (9.80, 12.57) | −4.38% |
| Female | 6.27 (3.33, 9.01) | 4.41 (2.25, 6.35) | −29.67% |  | 5.94 (5.12, 6.84) | 5.76 (5.03, 6.49) | −3.03% |
| SDI region |  |  |  |  |  |  |  |
| High SDI | 16.45 (9.52, 23.23) | 9.51 (5.11, 13.69) | −42.19% |  | 8.06 (7.32, 8.75) | 7.44 (6.55, 8.30) | −7.69% |
| High-middle SDI | 15.00 (8.66, 20.43) | 9.68 (5.44, 13.53) | −35.47% |  | 7.60 (6.90, 8.32) | 6.72 (5.78, 7.71) | −11.58% |
| Middle SDI | 11.97 (6.87, 16.11) | 9.39 (5.24, 12.82) | −21.55% |  | 7.81 (7.02, 8.62) | 7.51 (6.75, 8.34) | −3.84% |
| Low-middle SDI | 13.88 (7.49, 19.13) | 10.45 (5.34, 14.66) | −24.71% |  | 9.83 (8.14, 11.56) | 8.71 (7.41, 10.07) | −11.39% |
| Low SDI | 8.67 (4.47, 12.31) | 7.29 (3.64, 10.44) | −15.92% |  | 11.22 (9.57, 13.07) | 10.04 (8.71, 11.46) | −10.52% |
| **Abbreviations:** DALYs, disability adjusted life year; SDI, socio-demographic index. | | | | | | | |

| **Table S10:** Net and local drifts for asthma deaths and DALYs rates attributed to smoking (%). | | | | | | | | | | | | | | | | | | | | | | | | | | | | | | | | | |  | |  |  |
| --- | --- | --- | --- | --- | --- | --- | --- | --- | --- | --- | --- | --- | --- | --- | --- | --- | --- | --- | --- | --- | --- | --- | --- | --- | --- | --- | --- | --- | --- | --- | --- | --- | --- | --- | --- | --- | --- |
|  | |  | | Global | | | |  | | High SDI | | |  | | High-middle SDI | | |  | | | Middle SDI | | |  | | Low-middle SDI | | | |  | | Low SDI | | | |  |  |
|  | |  | | Deaths rate | | DALYs rate | |  | | Deaths rate | DALYs rate | |  | | Deaths rate | | DALYs rate |  | | | Deaths rate | DALYs rate | |  | | Deaths rate | | DALYs rate | |  | | Deaths rate | | DALYs rate | |  |  |
| Sex | | Net drifts (95% *CI*) | | | | | | | | | | | | | | | | | | | | | | | | | | | | | | | | | |  |  |
| Both | |  | | −3.54 (−3.61, −3.47) | | −3.34 (−3.40, −3.28) | |  | | −6.69 (−6.86, −6.52) | −3.93 (−4.04, −3.82) | |  | | −5.60 (−5.79, −5.41) | | −4.41 (−4.51, −4.31) |  | | | −3.35 (−3.43, −3.27) | −3.01 (−3.07, −2.95) | |  | | −3.51 (−3.63, −3.39) | | −3.29 (−3.40, −3.18) | |  | | −2.52 (−2.64, −2.40) | | −2.36 (−2.43, −2.29) | |  |  |
| Male | |  | | −3.66 (−3.73, −3.59) | | −3.50 (−3.56, −3.45) | |  | | −7.44 (−7.65, −7.24) | −4.89 (−4.98, −4.79) | |  | | −5.86 (−6.08, −5.64) | | −4.92 (−5.03, −4.80) |  | | | −3.29 (−3.37, −3.21) | −2.95 (−3.02, −2.88) | |  | | −3.44 (−3.57, −3.32) | | −3.22 (−3.33, −3.10) | |  | | −2.70 (−2.84, −2.56) | | −2.53 (−2.61, −2.45) | |  |  |
| Female | |  | | −3.42 (−3.53, −3.32) | | −3.06 (−3.15, −2.98) | |  | | −5.71 (−5.96, −5.45) | −2.96 (−3.12, −2.80) | |  | | −5.08 (−5.38, −4.78) | | −3.38 (−3.45, −3.31) |  | | | −3.74 (−3.98, −3.50) | −3.37 (−3.44, −3.29) | |  | | −3.19 (−3.39, −2.99) | | −3.02 (−3.17, −2.86) | |  | | −1.75 (−2.01, −1.49) | | −1.65 (−1.72, −1.57) | |  |  |
| Age group | | Local drifts (95% *CI*) | | | | | | | | | | | | | | | | | | | | | | | | | | | | | | | | | |  |  |
| 30-34 | |  | | −3.22 (−3.65, −2.79) | | −2.60 (−2.77, −2.42) | |  | | −3.69 (−4.60, −2.78) | −1.90 (−2.18, −1.61) | |  | | −5.15 (−6.47, −3.81) | | −2.62 (−2.89, −2.35) |  | | | −2.68 (−3.14, −2.21) | −1.99 (−2.17, −1.81) | |  | | −4.09 (−4.92, −3.25) | | −3.42 (−3.83, −3.00) | |  | | −2.40 (−3.09, −1.72) | | −2.03 (−2.23, −1.83) | |  |  |
| 35-39 | |  | | −3.19 (−3.47, −2.91) | | −2.69 (−2.81, −2.56) | |  | | −4.09 (−4.71, −3.46) | −1.93 (−2.14, −1.72) | |  | | −5.36 (−6.20, −4.52) | | −2.96 (−3.16, −2.77) |  | | | −2.76 (−3.06, −2.45) | −2.19 (−2.32, −2.06) | |  | | −3.85 (−4.37, −3.34) | | −3.35 (−3.63, −3.07) | |  | | −2.51 (−2.96, −2.06) | | −2.18 (−2.32, −2.04) | |  |  |
| 40-44 | |  | | −3.36 (−3.57, −3.15) | | −2.94 (−3.04, −2.83) | |  | | −4.50 (−5.00, −4.00) | −2.07 (−2.26, −1.89) | |  | | −5.81 (−6.43, −5.18) | | −3.65 (−3.81, −3.48) |  | | | −3.08 (−3.31, −2.84) | −2.53 (−2.64, −2.42) | |  | | −3.73 (−4.10, −3.36) | | −3.33 (−3.55, −3.12) | |  | | −2.70 (−3.04, −2.36) | | −2.40 (−2.51, −2.28) | |  |  |
| 45-49 | |  | | −3.66 (−3.83, −3.5) | | −3.21 (−3.30, −3.12) | |  | | −4.99 (−5.40, −4.58) | −2.31 (−2.49, −2.14) | |  | | −6.11 (−6.60, −5.62) | | −4.21 (−4.36, −4.05) |  | | | −3.22 (−3.41, −3.03) | −2.75 (−2.84, −2.65) | |  | | −3.74 (−4.03, −3.46) | | −3.42 (−3.60, −3.24) | |  | | −2.86 (−3.13, −2.59) | | −2.60 (−2.70, −2.50) | |  |  |
| 50-54 | |  | | −3.72 (−3.85, −3.58) | | −3.31 (−3.39, −3.22) | |  | | −5.70 (−6.05, −5.35) | −2.74 (−2.91, −2.57) | |  | | −6.32 (−6.71, −5.93) | | −4.59 (−4.73, −4.44) |  | | | −3.19 (−3.35, −3.03) | −2.83 (−2.92, −2.74) | |  | | −3.59 (−3.81, −3.36) | | −3.35 (−3.50, −3.19) | |  | | −2.82 (−3.05, −2.6) | | −2.61 (−2.70, −2.53) | |  |  |
| **Table S10:** Continued. | | | | | | | | | | | | | | | | | | | | | | | | | | | | | | | | | | | | | |
| ` |  | | Global | | | |  | | High SDI | | |  | | High-middle SDI | | | | |  | Middle SDI | | | | |  | | Low-middle SDI | | | |  | | Low SDI | | | |  |
|  |  | | Deaths rate | | DALYs rate | |  | | Deaths rate | | DALYs rate |  | | Deaths rate | | DALYs rate | | |  | Deaths rate | | | DALYs rate | |  | | Deaths rate | | DALYs rate | |  | | Deaths rate | | DALYs rate | |  |
| 55-59 |  | | −3.84 (−3.96, −3.73) | | −3.49 (−3.57, −3.41) | |  | | −6.75 (−7.06, −6.43) | | −3.39 (−3.55, −3.22) |  | | −6.43 (−6.75, −6.11) | | −4.90 (−5.04, −4.77) | | |  | −3.28 (−3.41, −3.14) | | | −2.99 (−3.08, −2.91) | |  | | −3.64 (−3.82, −3.45) | | −3.43 (−3.57, −3.29) | |  | | −2.80 (−2.99, −2.61) | | −2.63 (−2.71, −2.55) | |  |
| 60-64 |  | | −3.92 (−4.02, −3.82) | | −3.71 (−3.78, −3.63) | |  | | −7.87 (−8.16, −7.59) | | −4.24 (−4.41, −4.07) |  | | −6.35 (−6.62, −6.07) | | −5.16 (−5.29, −5.02) | | |  | −3.63 (−3.75, −3.51) | | | −3.35 (−3.43, −3.27) | |  | | −3.79 (−3.95, −3.63) | | −3.60 (−3.73, −3.47) | |  | | −2.67 (−2.84, −2.5) | | −2.55 (−2.63, −2.47) | |  |
| 65-69 |  | | −3.76 (−3.85, −3.67) | | −3.70 (−3.78, −3.62) | |  | | −8.58 (−8.85, −8.32) | | −5.02 (−5.19, −4.84) |  | | −5.88 (−6.13, −5.63) | | −5.05 (−5.19, −4.91) | | |  | −3.76 (−3.87, −3.65) | | | −3.49 (−3.57, −3.4) | |  | | −3.75 (−3.90, −3.60) | | −3.59 (−3.72, −3.45) | |  | | −2.51 (−2.68, −2.35) | | −2.42 (−2.50, −2.33) | |  |
| 70-74 |  | | −3.60 (−3.69, −3.51) | | −3.68 (−3.78, −3.59) | |  | | −8.63 (−8.87, −8.38) | | −5.62 (−5.81, −5.42) |  | | −5.47 (−5.71, −5.22) | | −4.90 (−5.06, −4.74) | | |  | −3.76 (−3.87, −3.65) | | | −3.50 (−3.60, −3.41) | |  | | −3.50 (−3.65, −3.35) | | −3.38 (−3.53, −3.23) | |  | | −2.33 (−2.50, −2.16) | | −2.25 (−2.34, −2.15) | |  |
| 75-79 |  | | −3.43 (−3.53, −3.33) | | −3.59 (−3.7, −3.48) | |  | | −8.29 (−8.52, −8.05) | | −5.87 (−6.10, −5.65) |  | | −4.90 (−5.15, −4.65) | | −4.60 (−4.79, −4.41) | | |  | −3.63 (−3.74, −3.52) | | | −3.40 (−3.51, −3.29) | |  | | −3.25 (−3.43, −3.08) | | −3.18 (−3.37, −2.98) | |  | | −2.22 (−2.42, −2.02) | | −2.15 (−2.28, −2.02) | |  |
| 80-84 |  | | −3.17 (−3.29, −3.05) | | −3.32 (−3.47, −3.16) | |  | | −7.53 (−7.78, −7.28) | | −5.58 (−5.88, −5.29) |  | | −4.50 (−4.79, −4.21) | | −4.31 (−4.57, −4.05) | | |  | −3.33 (−3.47, −3.19) | | | −3.12 (−3.28, −2.96) | |  | | −2.82 (−3.04, −2.6) | | −2.77 (−3.06, −2.49) | |  | | −2.14 (−2.40, −1.87) | | −2.08 (−2.28, −1.89) | |  |
| 85-89 |  | | −3.07 (−3.25, −2.88) | | −3.07 (−3.34, −2.81) | |  | | −6.43 (−6.74, −6.12) | | −4.75 (−5.22, −4.28) |  | | −4.12 (−4.54, −3.7) | | −3.93 (−4.37, −3.49) | | |  | −3.16 (−3.37, −2.96) | | | −2.97 (−3.24, −2.70) | |  | | −2.62 (−2.98, −2.26) | | −2.56 (−3.08, −2.04) | |  | | −2.15 (−2.59, −1.71) | | −2.10 (−2.47, −1.73) | |  |
| 90-94 |  | | −2.96 (−3.32, −2.59) | | −2.82 (−3.42, −2.21) | |  | | −5.37 (−5.90, −4.84) | | −3.74 (−4.70, −2.76) |  | | −4.03 (−4.87, −3.18) | | −3.70 (−4.73, −2.67) | | |  | −3.03 (−3.46, −2.60) | | | −2.84 (−3.46, −2.21) | |  | | −2.52 (−3.26, −1.78) | | −2.45 (−3.66, −1.23) | |  | | −2.05 (−2.92, −1.18) | | −2.01 (−2.82, −1.19) | |  |
| **Notes:** *CI,* confidence interval. Net drifts represent the overall annual percentage change in the age-standardized rate based on period and birth cohort. Local drifts indicate the annual percentage change over time specific to the age group. All of net and local drifts were statistically significant (*P*<0.05). | | | | | | | | | | | | | | | | | | | | | | | | | | | | | | | | | | | | | |

| **Table S11:** Net and local drifts for asthma deaths and DALYs rates attributed to occupational asthmagens (%). | | | | | | | | | | | | | | | | | | | | | | | |  |  |
| --- | --- | --- | --- | --- | --- | --- | --- | --- | --- | --- | --- | --- | --- | --- | --- | --- | --- | --- | --- | --- | --- | --- | --- | --- | --- |
|  |  | Global | |  | High SDI | |  | High-middle SDI | |  | Middle SDI | |  | Low-middle SDI | | |  | | Low SDI | | | |  |  |  |
|  |  | Deaths rate | DALYs rate |  | Deaths rate | DALYs rate |  | Deaths rate | DALYs rate |  | Deaths rate | DALYs rate |  | Deaths rate | DALYs rate | |  | | Deaths rate | | DALYs rate | |  |  |  |
| Sex | Net drifts (95% *CI*) | | | | | | | | | | | | | | | | | | | | | | |  |  |
| Both |  | −2.60 (−2.68, −2.53) | −2.31 (−2.37, −2.26) |  | −4.99 (−5.27, −4.70) | −2.24 (−2.35, −2.12) |  | −4.84 (−5.07, −4.6) | −3.53 (−3.61, −3.46) |  | −2.87 (−2.98, −2.75) | −2.42 (−2.49, −2.36) |  | −2.84 (−2.97, −2.72) | −2.53 (−2.61, −2.44) | |  | | −2.48 (−2.60, −2.37) | | −2.24 (−2.28, −2.21) | |  |  |  |
| Male |  | −2.62 (−2.71, −2.53) | −2.39 (−2.44, −2.33) |  | −5.40 (−5.75, −5.06) | −2.99 (−3.10, −2.89) |  | −4.92 (−5.20, −4.64) | −3.80 (−3.89, −3.7) |  | −2.71 (−2.84, −2.60) | −2.28 (−2.36, −2.21) |  | −2.68 (−2.83, −2.54) | −2.38 (−2.48, −2.28) | |  | | −2.50 (−2.65, −2.36) | | −2.26 (−2.32, −2.20) | |  |  |  |
| Female |  | −2.58 (−2.68, −2.48) | −2.18 (−2.24, −2.13) |  | −4.22 (−4.74, −3.7) | −1.17 (−1.34, −1.01) |  | −4.75 (−5.18, −4.33) | −3.14 (−3.24, −3.04) |  | −3.19 (−3.40, −2.97) | −2.68 (−2.76, −2.60) |  | −3.07 (−3.24, −2.91) | −2.74 (−2.83, −2.65) | |  | | −2.38 (−2.56, −2.20) | | −2.15 (−2.20, −2.10) | |  |  |  |
| Age group | Local drifts (95% *CI*) | | | | | | | | | | | | | | | | | | | | | |  |  |  |
| 15-19 |  | −2.12 (−2.69, −1.53) | −1.53 (−1.74, −1.33) |  | −4.90 (−7.25, −2.49) | −1.83 (−2.22, −1.45) |  | −5.20 (−7.63, −2.71) | −2.75 (−3.09, −2.41) |  | −2.08 (−3.02, −1.12) | −1.43 (−1.70, −1.16) |  | −3.03 (−4.03, −2.03) | −1.80 (−2.23, −1.38) | |  | | −2.54 (−3.19, −1.88) | | −1.84 (−1.96, −1.73) | |  |  |  |
| 20-24 |  | −2.09 (−2.43, −1.75) | −1.59 (−1.73, −1.45) |  | −3.91 (−5.12, −2.68) | −1.60 (−1.84, −1.36) |  | −4.72 (−5.89, −3.54) | −2.38 (−2.58, −2.18) |  | −2.05 (−2.56, −1.53) | −1.36 (−1.53, −1.18) |  | −2.72 (−3.30, −2.13) | −1.87 (−2.16, −1.58) | |  | | −2.45 (−2.88, −2.02) | | −1.97 (−2.06, −1.88) | |  |  |  |
| 25-29 |  | −2.10 (−2.36, −1.84) | −1.63 (−1.75, −1.52) |  | −3.41 (−4.27, −2.55) | −1.34 (−1.53, −1.14) |  | −4.50 (−5.27, −3.73) | −2.25 (−2.4, −2.10) |  | −2.27 (−2.64, −1.90) | −1.50 (−1.64, −1.36) |  | −2.59 (−3.02, −2.16) | −1.97 (−2.21, −1.74) | |  | | −2.35 (−2.7, −2.01) | | −1.96 (−2.04, −1.88) | |  |  |  |
| 30-34 |  | −2.04 (−2.26, −1.83) | −1.68 (−1.78, −1.57) |  | −3.29 (−3.99, −2.59) | −1.21 (−1.38, −1.03) |  | −4.32 (−4.93, −3.7) | −2.31 (−2.44, −2.17) |  | −2.31 (−2.61, −2.00) | −1.63 (−1.76, −1.5) |  | −2.58 (−2.93, −2.23) | −2.10 (−2.31, −1.9) | |  | | −2.33 (−2.63, −2.03) | | −2.00 (−2.07, −1.92) | |  |  |  |
| 35-39 |  | −2.12 (−2.30, −1.94) | −1.81 (−1.91, −1.72) |  | −3.50 (−4.10, −2.89) | −1.31 (−1.48, −1.14) |  | −4.48 (−4.98, −3.97) | −2.64 (−2.77, −2.52) |  | −2.32 (−2.57, −2.06) | −1.74 (−1.86, −1.62) |  | −2.61 (−2.90, −2.32) | −2.23 (−2.41, −2.04) | |  | | −2.38 (−2.65, −2.12) | | −2.08 (−2.15, −2.00) | |  |  |  |
| **Table S11:** Continued. | | | | | | | | | | | | | | | | | | | | | | | | | |
|  |  | Global | |  | High SDI | |  | High-middle SDI | |  | Middle SDI | |  | Low-middle SDI | | | |  | | Low SDI | | | | |  |
|  |  | Deaths rate | DALYs rate |  | Deaths rate | DALYs rate |  | Deaths rate | DALYs rate |  | Deaths rate | DALYs rate |  | Deaths rate | | DALYs rate | |  | | Deaths rate | | DALYs rate | | |  |
| 40-44 |  | −2.38 (−2.54, −2.23) | −2.10 (−2.19, −2.01) |  | −3.74 (−4.27, −3.2) | −1.47 (−1.63, −1.3) |  | −4.85 (−5.28, −4.42) | −3.22 (−3.34, −3.10) |  | −2.57 (−2.79, −2.35) | −2.04 (−2.15, −1.92) |  | −2.71 (−2.95, −2.46) | | −2.39 (−2.56, −2.22) | |  | | −2.48 (−2.71, −2.25) | | −2.21 (−2.28, −2.14) | | |  |
| 45-49 |  | −2.87 (−3.00, −2.73) | −2.53 (−2.62, −2.44) |  | −4.23 (−4.70, −3.75) | −1.69 (−1.85, −1.52) |  | −5.22 (−5.58, −4.85) | −3.75 (−3.87, −3.63) |  | −2.82 (−3.01, −2.62) | −2.35 (−2.47, −2.24) |  | −2.90 (−3.10, −2.69) | | −2.63 (−2.79, −2.48) | |  | | −2.63 (−2.83, −2.43) | | −2.39 (−2.45, −2.32) | | |  |
| 50-54 |  | −3.00 (−3.11, −2.88) | −2.69 (−2.77, −2.6) |  | −4.92 (−5.36, −4.48) | −1.95 (−2.12, −1.78) |  | −5.35 (−5.67, −5.03) | −4.09 (−4.21, −3.97) |  | −2.87 (−3.05, −2.7) | −2.51 (−2.63, −2.4) |  | −2.85 (−3.03, −2.67) | | −2.68 (−2.82, −2.53) | |  | | −2.66 (−2.84, −2.48) | | −2.46 (−2.52, −2.40) | | |  |
| 55-59 |  | −3.11 (−3.22, −3.00) | −2.83 (−2.91, −2.74) |  | −5.74 (−6.17, −5.30) | −2.32 (−2.52, −2.13) |  | −5.30 (−5.59, −5.01) | −4.28 (−4.40, −4.15) |  | −3.01 (−3.17, −2.85) | −2.72 (−2.83, −2.6) |  | −2.98 (−3.14, −2.82) | | −2.83 (−2.96, −2.69) | |  | | −2.68 (−2.85, −2.52) | | −2.52 (−2.58, −2.46) | | |  |
| 60-64 |  | −3.11 (−3.21, −3.01) | −2.89 (−2.98, −2.79) |  | −6.48 (−6.94, −6.02) | −2.81 (−3.04, −2.58) |  | −5.04 (−5.33, −4.75) | −4.35 (−4.50, −4.21) |  | −3.40 (−3.56, −3.24) | −3.11 (−3.24, −2.99) |  | −3.20 (−3.34, −3.05) | | −3.05 (−3.19, −2.91) | |  | | −2.66 (−2.82, −2.5) | | −2.53 (−2.59, −2.46) | | |  |
| 65-69 |  | −2.87 (−2.98, −2.76) | −2.71 (−2.82, −2.60) |  | −6.93 (−7.45, −6.4) | −3.34 (−3.65, −3.03) |  | −4.67 (−4.99, −4.35) | −4.25 (−4.44, −4.07) |  | −3.56 (−3.72, −3.39) | −3.29 (−3.44, −3.14) |  | −3.18 (−3.34, −3.02) | | −3.04 (−3.21, −2.88) | |  | | −2.55 (−2.72, −2.38) | | −2.43 (−2.50, −2.35) | | |  |
| 70-74 |  | −2.74 (−2.86, −2.61) | −2.65 (−2.79, −2.50) |  | −7.08 (−7.66, −6.49) | −4.05 (−4.46, −3.64) |  | −4.55 (−4.91, −4.18) | −4.25 (−4.49, −4.01) |  | −3.65 (−3.84, −3.46) | −3.40 (−3.59, −3.21) |  | −2.99 (−3.18, −2.8) | | −2.87 (−3.09, −2.65) | |  | | −2.35 (−2.55, −2.15) | | −2.25 (−2.35, −2.15) | | |  |
| 75-79 |  | −2.58 (−2.75, −2.41) | −2.50 (−2.72, −2.29) |  | −6.75 (−7.43, −6.07) | −4.29 (−4.86, −3.73) |  | −4.32 (−4.76, −3.87) | −4.04 (−4.38, −3.71) |  | −3.71 (−3.96, −3.46) | −3.48 (−3.75, −3.2) |  | −2.79 (−3.05, −2.54) | | −2.70 (−3.03, −2.36) | |  | | −2.16 (−2.44, −1.88) | | −2.08 (−2.24, −1.93) | | |  |
| **Notes:** *CI,* confidence interval. Net drifts represent the overall annual percentage change in the age-standardized rate based on period and birth cohort. Local drifts indicate the annual percentage change over time specific to the age group. All of net and local drifts were statistically significant (*P*<0.05). | | | | | | | | | | | | | | | | | | | | | | | | | |

**References**

1. Safiri S, Carson-Chahhoud K, Karamzad N, Sullman MJM, Nejadghaderi SA, Taghizadieh A, et al. Prevalence, Deaths, and Disability-Adjusted Life-Years Due to Asthma and Its Attributable Risk Factors in 204 Countries and Territories, 1990-2019. Chest. 2022;161(2):318-29.
2. Kennedy SM, Le Moual N, Choudat D, Kauffmann F. Development of an asthma specific job exposure matrix and its application in the epidemiological study of genetics and environment in asthma (EGEA). Occupational and environmental medicine. 2000;57(9):635-41.

**Figure S1:** Age-standardized male asthma deaths and DALYs rates attributed to different risk factors in 2019 by countries.

(A) Age-standardized asthma deaths rates attributed to smoking for male; (B) Age-standardized asthma DALYs rates attributed to smoking for male; (C) Age-standardized asthma deaths rates attributed to occupational asthmagens for male; (D) Age-standardized asthma DALYs rates attributed to occupational asthmagens for male.


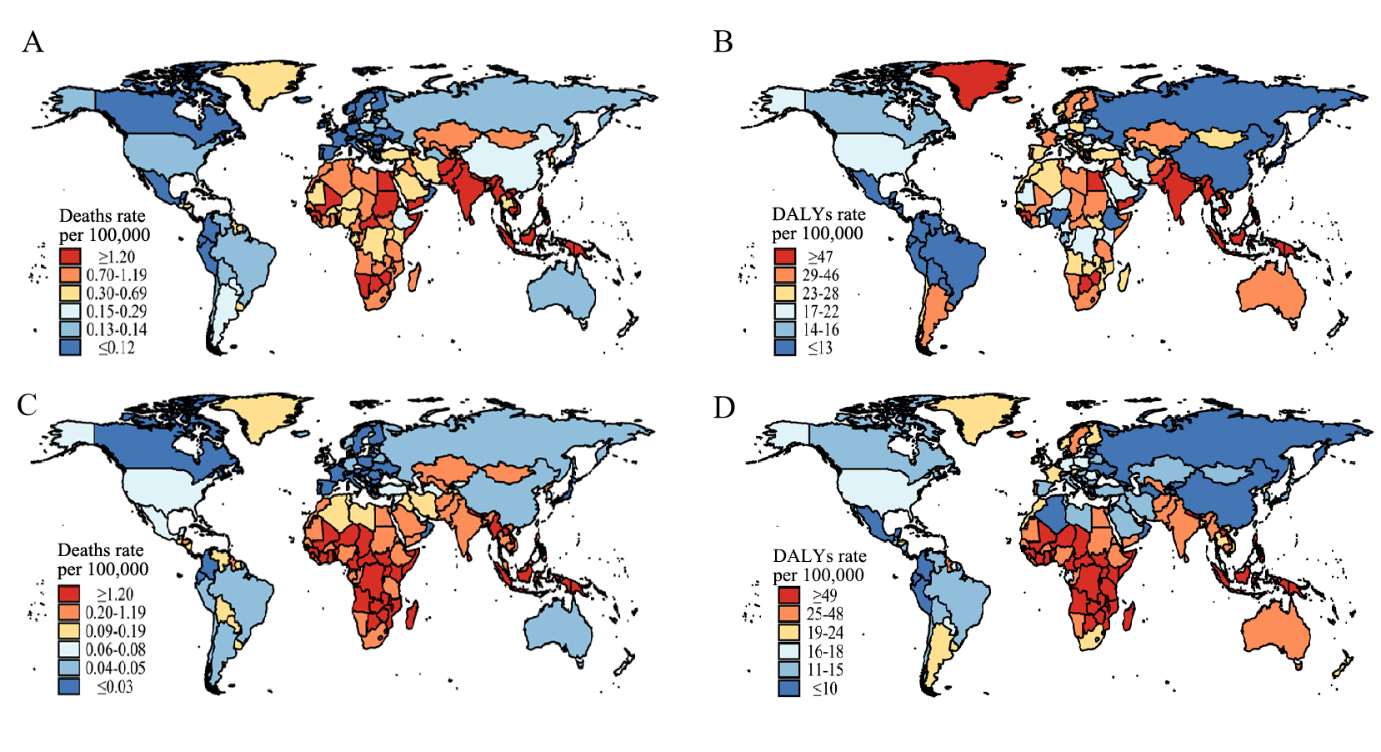


**Figure S2:** Age-standardized female asthma deaths and DALYs rates attributed to different risk factors in 2019 by countries.

(A) Age-standardized asthma deaths rates attributed to smoking for female; (B) Age-standardized asthma DALYs rates attributed to smoking for female; (C) Age-standardized asthma deaths rates attributed to occupational asthmagens for female; (D) Age-standardized asthma DALYs rates attributed to occupational asthmagens for female.


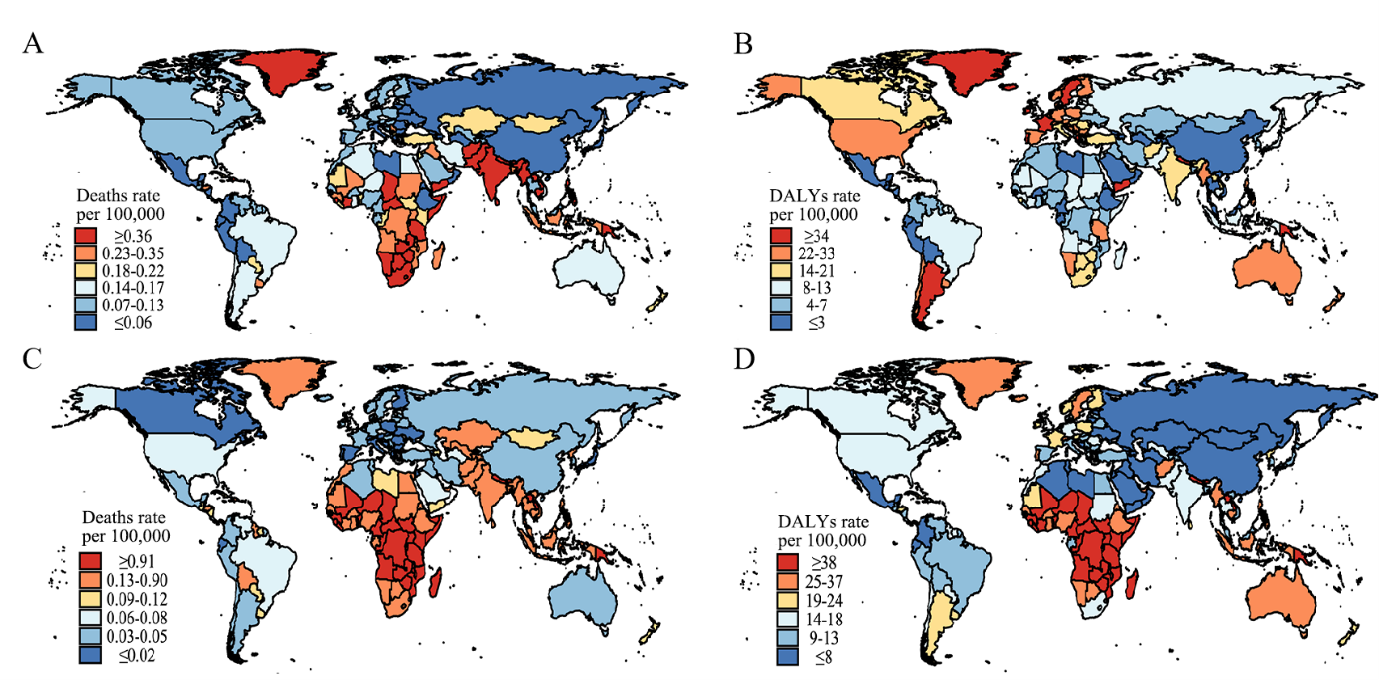


**Figure S3:** Age, period, and cohort effects on asthma deaths and DALYs rates attributed to smoking for male.

(A) Age-specific asthma deaths rates attributed to smoking for male; (B) Age-specific asthma DALYs rates attributed to smoking for male; (C) Period rate ratio (RR) of asthma deaths rates attributed to smoking for male; (D) Period RR of asthma DALYs rates attributed to smoking for male; (E) Cohort RR of asthma deaths rates attributed to smoking for male; (F) Cohort RR of asthma DALYs rates attributed to smoking for male.


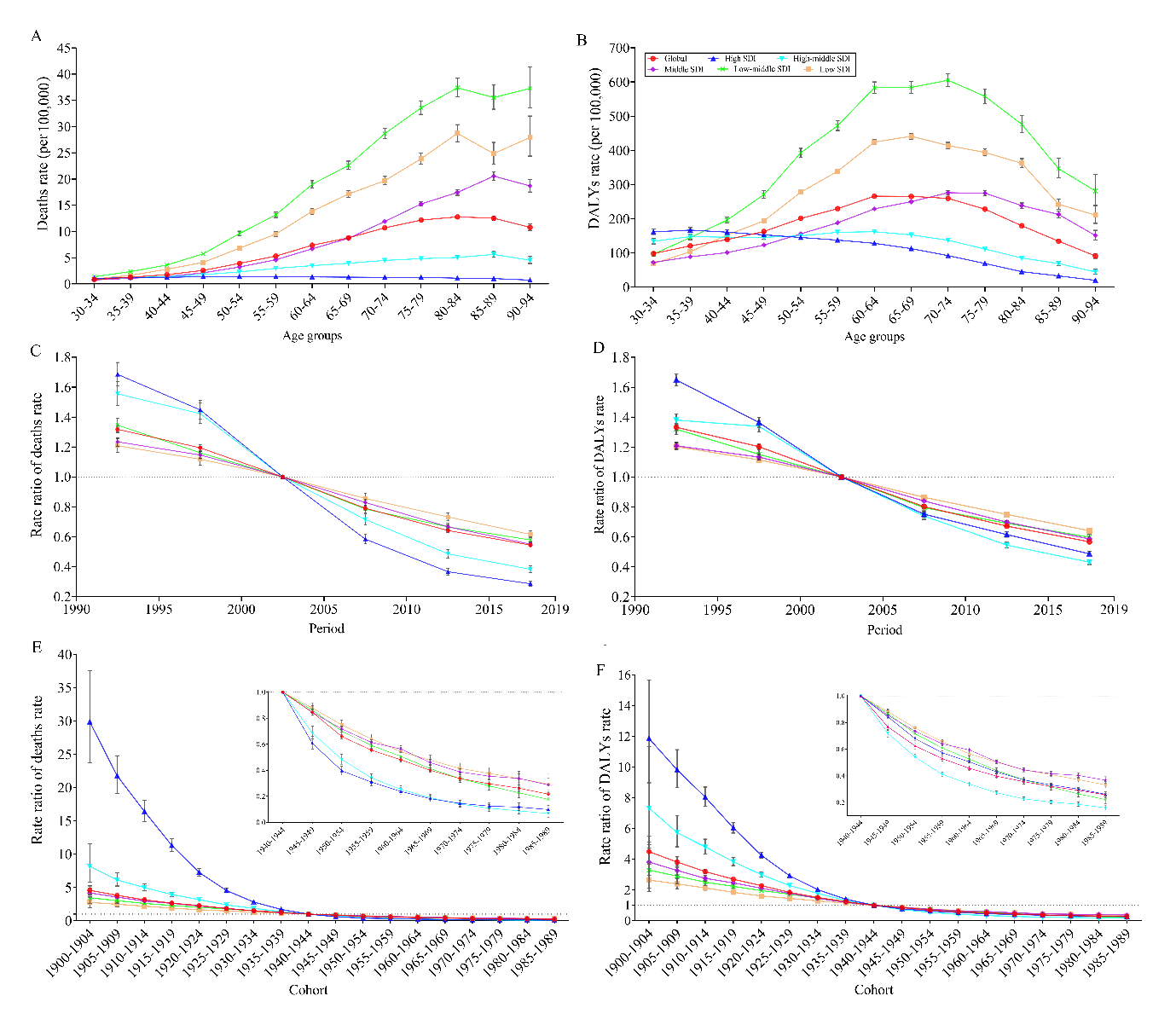


**Figure S4:** Age, period, and cohort effects on asthma deaths and DALYs rates attributed to smoking for female.

(A) Age-specific asthma deaths rates attributed to smoking for female; (B) Age-specific asthma DALYs rates attributed to smoking for female; (C) Period rate ratio (RR) of asthma deaths rates attributed to smoking for female; (D) Period RR of asthma DALYs rates attributed to smoking for female; (E) Cohort RR of asthma deaths rates attributed to smoking for female; (F) Cohort RR of asthma DALYs rates attributed to smoking for female.


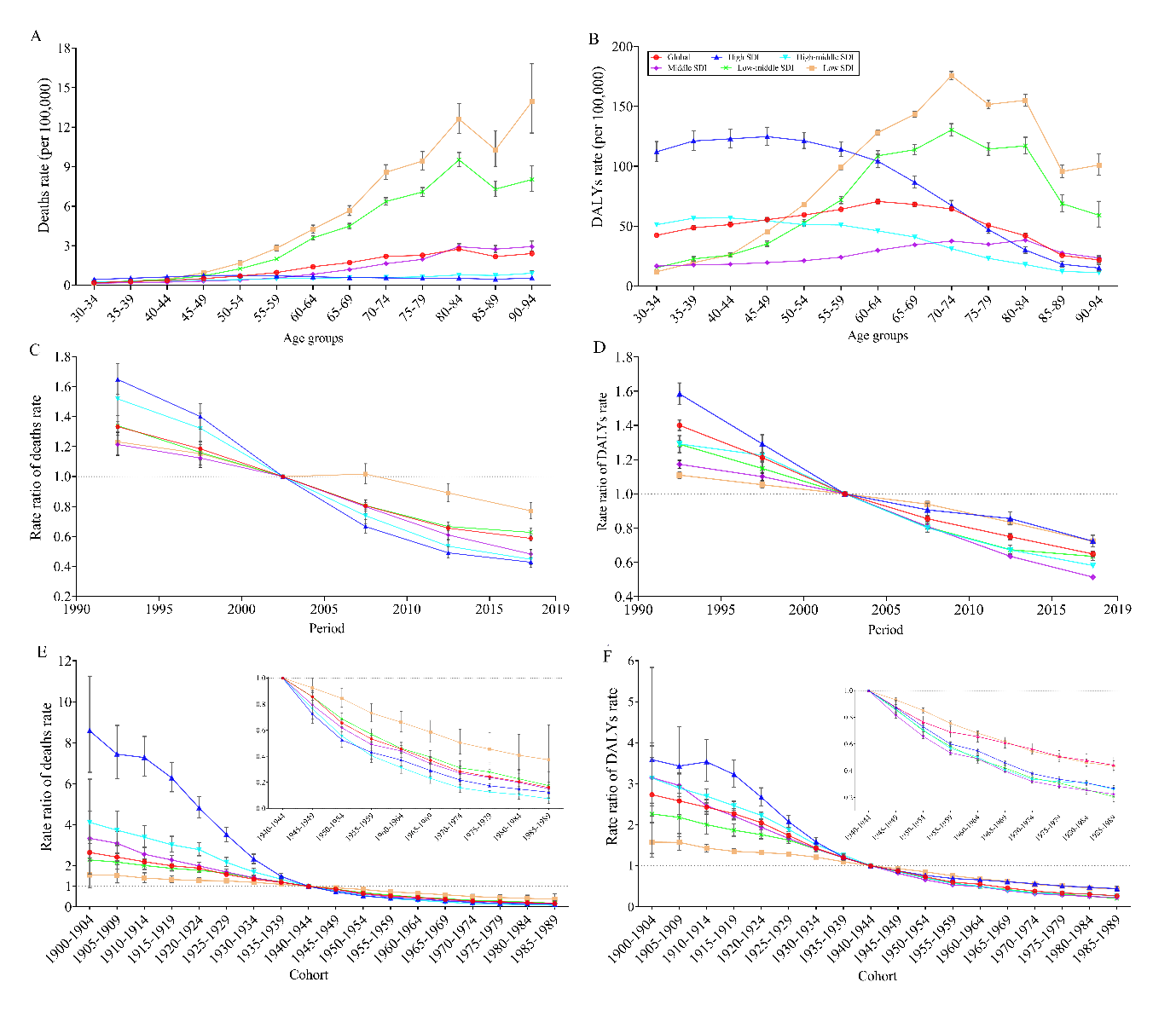


**Figure S5:** Age, period, and cohort effects on asthma deaths and DALYs rates attributed to occupational asthmagens for male.

(A) Age-specific asthma deaths rates attributed to occupational asthmagens for male; (B) Age-specific asthma DALYs rates attributed to occupational asthmagens for male; (C) Period rate ratio (RR) of asthma deaths rates attributed to asthmagens for male; (D) Period RR of asthma DALYs rates attributed to occupational asthmagens for male; (E) Cohort RR of asthma deaths rates attributed to occupational asthmagens for male; (F) Cohort RR of asthma DALYs rates attributed to occupational asthmagens for male.


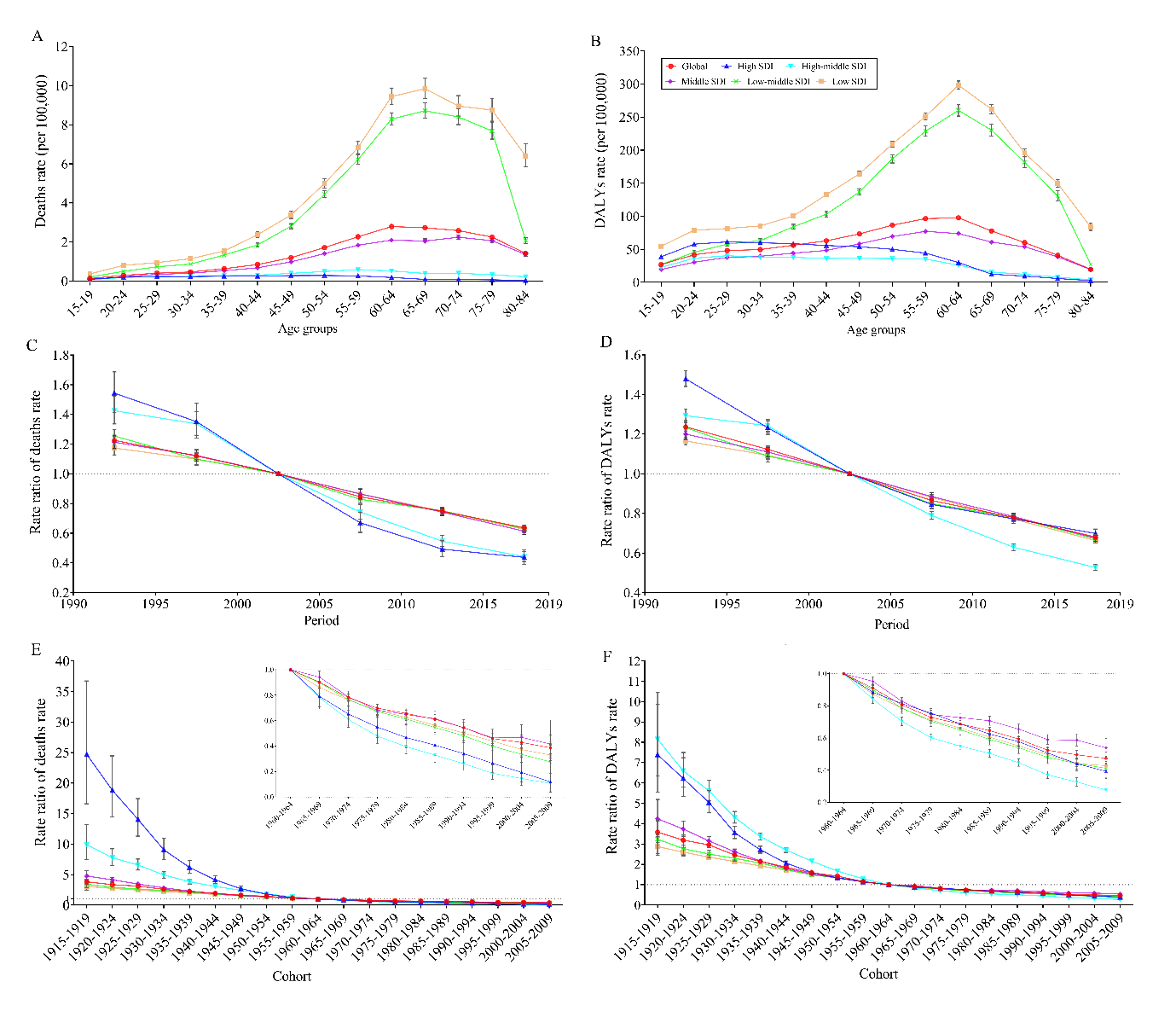


**Figure S6:** Age, period, and cohort effects on asthma deaths and DALYs rates attributed to occupational asthmagens for female.

(A) Age-specific asthma deaths rates attributed to occupational asthmagens for female; (B) Age-specific asthma DALYs rates attributed to occupational asthmagens for female; (C) Period rate ratio (RR) of asthma deaths rates attributed to asthmagens for female; (D) Period RR of asthma DALYs rates attributed to occupational asthmagens for female; (E) Cohort RR of asthma deaths rates attributed to occupational asthmagens for female; (F) Cohort RR of asthma DALYs rates attributed to occupational asthmagens for female.


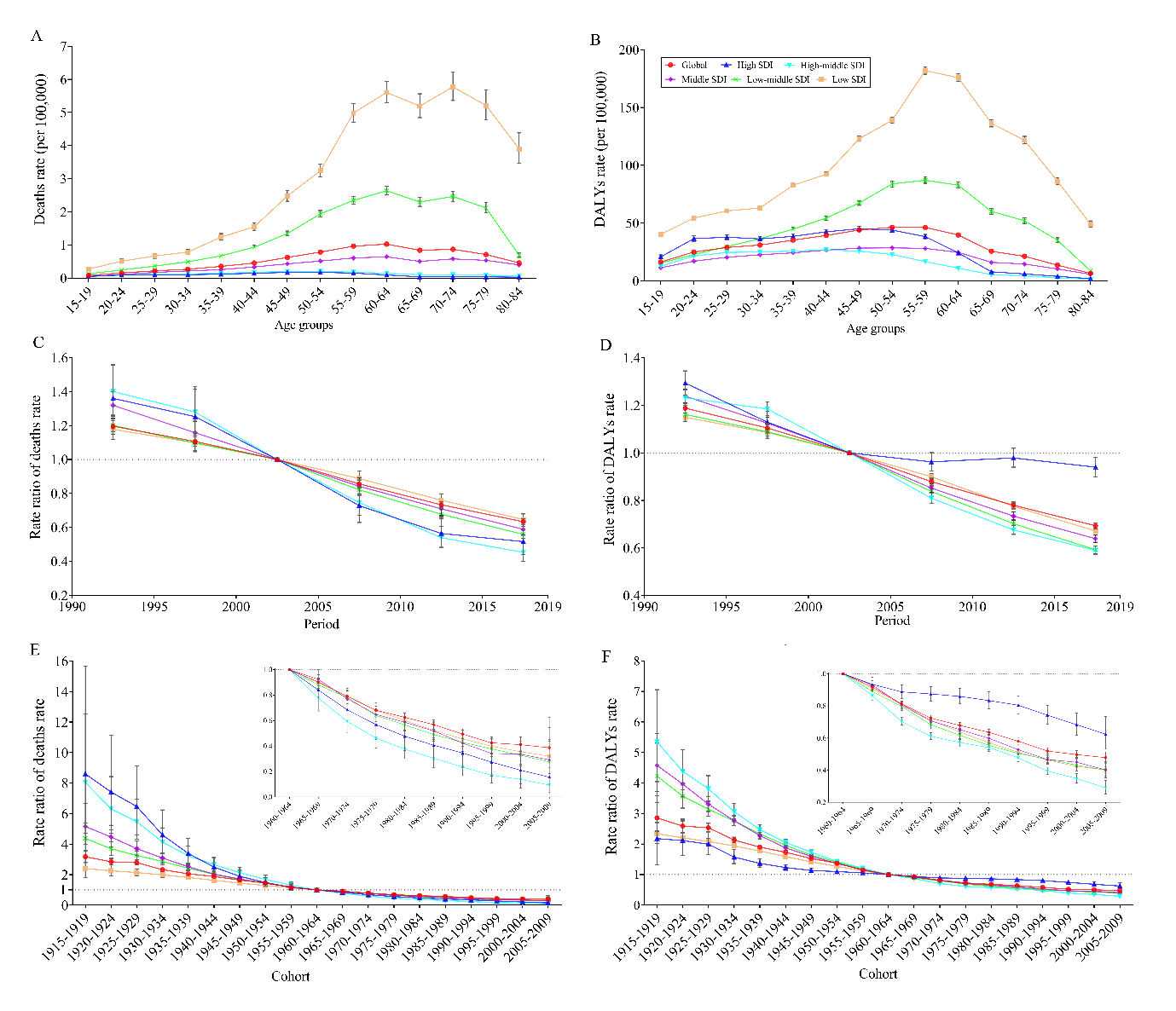

Supplement: Supplementary file 1 — Additional file 1: Table S1. Quintiles of different SDI groups. Table S2. Countries and territories of different SDI groups in 1990 and 2019. Table S3. Full list of selected covariates for the CODEm models in the asthma estimation. Table S4. Lay description, disability weight and proportion of different levels of asthma severity. Table S5. 22 occupational asthmagens recorded in the International Labor Organization. Table S6. Change in age-standardized asthma deaths number related to different risk factors from 1990 to 2019. Table S7. Change in age-standardized asthma deaths percent related to different risk factors from 1990 to 2019. Table S8. Change in age-standardized asthma DALYs number related to different risk factors from 1990 to 2019. Table S9. Change in age-standardized asthma DALYs percent related to different risk factors from 1990 to 2019. Table S10. Net and local drifts for asthma deaths and DALYs rates attributed to smoking (%). Table S11. Net and local drifts for asthma deaths and DALYs rates attributed to occupational asthmagens (%). Figure S1. Age-standardized male asthma deaths and DALYs rates attributed to different risk factors in 2019 by countries. Figure S2. Age-standardized female asthma deaths and DALYs rates attributed to different risk factors in 2019 by countries. Figure S3. Age, period, and cohort effects on asthma deaths and DALYs rates attributed to smoking for male. Figure S4. Age, period, and cohort effects on asthma deaths and DALYs rates attributed to smoking for female. Figure S5. Age, period, and cohort effects on asthma deaths and DALYs rates attributed to occupational asthmagens for male. Figure S6. Age, period, and cohort effects on asthma deaths and DALYs rates attributed to occupational asthmagens for female. [file 12940_2024_1060_MOESM1_ESM.docx]
